# Utility of B-13 Progenitor-Derived Hepatocytes in Hepatotoxicity and Genotoxicity Studies

**DOI:** 10.1093/toxsci/kft258

**Published:** 2013-11-13

**Authors:** Philip M. E. Probert, Git W. Chung, Simon J. Cockell, Loranne Agius, Pasquale Mosesso, Steven A. White, Fiona Oakley, Colin D. A. Brown, Matthew C. Wright

**Affiliations:** *Institute of Cellular Medicine,; †Institute for Cell and Molecular Bioscience, and; ‡Bioinformatics Support Unit, Medical School, Newcastle University, Newcastle Upon Tyne NE2 4HH, UK; and; §Dipartimento di Agrobiologia e Agrochimica, Università degli Studi della Tuscia, 01100 Viterbo, Italy

**Keywords:** stem cell, liver, xenobiotic metabolism, cytochrome P450, transporters.

## Abstract

AR42J-B-13 (B-13) cells form hepatocyte-like (B-13/H) cells in response to glucocorticoid treatment. To establish its utility in toxicity and genotoxicity screening, cytochrome P450 (CYP) induction, susceptibility to toxins, and transporter gene expression were examined. Conversion to B-13/H cells resulted in expression of male-specific CYP2C11 and sensitivity to methapyrilene. B-13/H cells constitutively expressed CYP1A, induced expression in response to an aryl hydrocarbon receptor agonist, and activated benzo[α]pyrene to a DNA-damaging species. Functional CYP1A2 was not expressed due to deletions in the ***Cyp1a2*** gene. A B-13 cell line stably expressing the human CYP1A2 was therefore engineered (B-13^−TR/h1A2^) and the derived B-13/H cells expressed metabolically functional CYP1A2. Treatment with the cooked food mutagen 2-amino-1-methyl-6-phenylimidazo(4,5-b)pyridine resulted in a dose-dependent increase in DNA damage. B-13/H cells expressed constitutive androstane receptor (CAR) and induced CYP2B1 mRNA levels in response to classical CAR activators. However, translation to functional CYP2B1 protein was low and increased minimally by CAR activator treatment. B-13/H cells expressed high levels of pregnane X-receptor (PXR) and induced CYP3A1 in response to classical PXR activators. CYP3A genes were inducible, functional, and activated aflatoxin B1 to a DNA-damaging species. All 23 major hepatic transporters were induced when B-13 cells were converted to B-13/H cells, although in many cases, levels remained below those present in adult rat liver. However, bile salt export pump, Abcb1b, multidrug resistance-associated protein, and breast cancer resistance protein transporters were functional in B-13/H cells. These data demonstrate that the B-13 cell generates hepatocyte-like cells with functional drug metabolism and transporter activities, which can alone—or in a humanized form—be used to screen for hepatotoxic and genotoxic endpoints *in vitro*.

Hepatocytes are the primary defining cell of the liver, performing the vast majority of its functions (Wallace *et al.*, 2010a). Isolation and/or culture of hepatocytes are therefore common experimental techniques employed in the study of liver functions (Wallace *et al.*, 2010a). Several factors limit hepatocyte utility. Hepatocytes do not proliferate *in vitro* and therefore cannot be expanded *in vitro*. Furthermore, culture results in dedifferentiation and loss of function. Complex culture modifications can ameliorate this loss but experimentally introduce an array of often uncharacterized factors, which can complicate interpretation (Wallace *et al.*, 2010a).

In many cases, human hepatocytes would be the ideal species to use for most experiments related to toxicity. However, human liver is in short supply and is often of poor quality. In the absence of sufficient human hepatocytes from donor livers, the main alternative for generating human hepatocytes is through differentiation of human embryonic stem cells (ESCs) or induced pluripotent stem cells (iPSCs) (Lavon *et al.*, 2004; Rashid *et al.*, 2010; Takayama *et al.*, 2012; Zhang *et al.*, 2013). Despite significant progress over the last 12 years, it has not been possible to generate hepatocytes with function quantitatively similar to adult human hepatocytes (Takayama *et al.*, 2012). Stem cell–derived hepatocytes remain in a fetal state and cannot progress further unless transplanted *in vivo* (Lavon *et al.*, 2004; Rashid *et al.*, 2010; Takayama *et al.*, 2012; Yu *et al.*, 2012; Zhang *et al.*, 2013). Because normal hepatocytes dedifferentiate into a fetal state *in vitro* (even when present within culture tissue slices) (Wallace *et al.*, 2010a; Wright and Paine, 1992), it may be not be surprising that this barrier exists in stem cell–derived hepatocyte culture. In addition, a major hurdle to the use of ESC/iPSC-derived hepatocyte is their high cost of generation. As part of an European Commission–funded project, the cost of hepatocytes derived from iPSCs was calculated, taking into consideration the most recent research that compared drug metabolism activity in iPSC-derived hepatocytes with human hepatocytes (Takayama *et al.*, 2012). In order to obtain the hepatocytes, the iPSCs required a 4-stage differentiation protocol with a variety of recombinant growth factors. In addition, the cells were infected with adenovirus directed to overexpress 2 transcription factors. Considering growth factors alone (without taking into account the cost of culture media, virus production, culture ware, and the cost of failures), it was calculated that the cost of generating hepatocytes is approximately 5 million times greater than using glucocorticoid and a human cell line with B-13 properties (d-LIVER consortium, 2012). With current technologies, it is unlikely that ESC- or iPSC-derived hepatocytes will be a practical solution in routine *in vitro* toxicity testing.

The B-13 cell could offer a potential route to delivering a cost-effective, simple solution to the production of functional hepatocytes *in vitro*. This rat pancreatic acinar-like cell line is readily expandable in simple culture medium and in response to one simple glucocorticoid hormone differentiates into nonproliferative hepatocyte-like (B-13/H) cells (Shen *et al.*, 2000; Wallace *et al.*, 2010b). B-13 cells model a pathophysiological response of rodent and human acinar tissue to differentiate into hepatocytes both *in vitro* and *in vivo* on exposure to high levels of glucocorticoid (Fairhall *et al.*, 2013b; Wallace *et al.*, 2009, 2010c). The cells retain a degree of biological stability in that they have maintained a normal karyotype in terms of the number of chromosomes per cell (although with some cytogenetic abnormalities); do not grow in soft agar (in contrast to tumorigenic cells); and are able to selectively engraft into the pancreas and liver (Fairhall *et al.*, 2013a). Within the liver, B-13 cells differentiate into hepatocytes (Fairhall *et al.*, 2013a). The intrinsic value of this cell line is that it offers an unlimited and reproducible supply of hepatocytes *in vitro*, without the requirement for tissue donors. Given both the simplicity and costs of a B-13 approach to hepatocyte generation, it is likely that a human B-13 equivalent would have utility in both experimental studies (eg, toxicity screening) and clinical applications (eg, extracorporeal liver support).

In this paper, we demonstrate that B-13/H cells recapitulate the sexually dimorphic expression of cytochrome P450 (CYP)2C11; retain CYP1-3 family induction to classic inducers; and express variable levels of functional transporter expression. Stable transfection of the human CYP1A2 gene resulted in a transgenic cell line with active human protein that metabolized a probe substrate and the progenotoxin 2-amino-1-methyl-6-phenylimidazo(4,5-b)pyridine (PhIP) to a genotoxic product.

## MATERIALS AND METHODS

PhIP was generously provided by Professor Nigel Gooderham (Imperial College, London, UK). All other materials were purchased from Sigma-Aldrich (Poole, UK) unless otherwise stated.

### 

#### 

##### Cell culture.

All cells were cultured in low glucose (1g/l) Dulbecco’s Modified Eagle’s Medium containing 10% (vol/vol) fetal calf serum, 100 units/ml penicillin, 100 μg/ml streptomycin, and 0.584g/l l-glutamine. This medium contains cysteine (and its precursor methionine) as well as the nonessential amino acids glycine and glutamine and is therefore capable of supporting glutathione synthesis. B-13 cells (originally referred to as AR42J-B-13 cells by the laboratory that isolated the clone from the parent AR42J cell line; Mashima *et al.*, 1996) were grown at 37°C at 5% CO_2_ in air in a humidified incubator, typically in 6-well plates. In some cases, B-13 cells were cultured in smaller culture formats up to 96-well plates dependent on the finally assay procedure. The B-13 cells had a doubling time of 30±7.3h and were subcultured every 2–3 days by trypsinization or differentiated to B-13/H cells via treatment with 10nM dexamethasone (DEX) for 14 days, with media changes every 2–3 days. B-13 cells expressing the tetracycline repressor were generated through transfection of B-13 cells with pcDNA6/TR (Life Technologies) using effectene (Qiagen) as previously described (Wallace *et al.*, 2010c) and selecting stably transfected B-13 cells (B-13^−TR^) using blastocidin at 8 μg/ml (for approximately 2 weeks), followed by a maintenance concentration of 4 µg/ml.

Rat hepatocytes were isolated essentially as previously described (Haughton *et al.*, 2006) and cultured identically to B-13 cells without serum. All compounds were added to media from 1000-fold concentrated vehicle-solvated stocks.

For induction studies, cells were routinely dosed daily with inducing agents for 3 days, with a media change every day. Cells were harvested on day 4 (24h after the last treatment with inducer). Inducers were used at the following final concentrations: β-naphthoflavone (β-NF), 20μM; androstenol, 5μM; phenobarbital (PB), 1mM; 1,4-Bis-[2-(3,5-dichloropyridyloxy)]benzene, 3,3′,5,5′-Tetrachloro-1,4-bis(pyridyloxy)benzene (TCPOBOP), 1.5μM; DEX, 10μM; 5-pregnen-3β-ol-20-one-16α-carbonitrile (PCN), 2μM; bezafibrate, 250μM.

##### Transmission electron microscopy.

Cells were suspended in 10mM EDTA in PBS (137mM NaCl, 2.7mM KCl, 10mM phosphate, pH 7.4), pelleted, and then fixed in 2% glutaraldehyde in Sorenson’s phosphate buffer (0.1M sodium phosphate, pH 7.2). The fixed cells were then processed and embedded in resin by the Newcastle University Electron Microscopy Research Service. Samples were viewed using a Philips CM100 transmission electron microscope.

##### Reverse transcriptase-PCR.

TRIzol (Invitrogen, Paisley, UK) was used in the isolation of total RNA from tissue or cells following the manufacturer’s instructions. Reverse transcriptase-PCR (RT-PCR) was performed on total RNA essentially as previously described (Haughton *et al.*, 2006). SYBR green was used for quantitative RT-PCR using a 7500 Fast Applied Biosciences thermocycler. The primer sequences are given in Table 1. The drug transporter array (SABiosciences, Crawley, UK) was run using the RT^2^ First Strand Kit and RT^2^ SYBR Green qPCR Mastermix (SABiosciences) following the protocol recommended by the manufacturer.

**TABLE 1:** Primers Used for PCR

Oligo ID	Primer Sequence (5′-3′)		Comments
18s rRNA	US	CCCGAAGCGTTTACTTTGAA	Will amplify 136bp of rat 18s rRNA
	DS	CCCTCTTAATCATGGCCTCA	
AhR	US	TGGCTGTGATGCCAAAGGGCAG	Will amplify 100-bp fragment of rat AhR (NM_013149.2)
	DS	AAGCATGTCAGCGGCGTGGA	
AhRNT	US	GGTTTGCCAGGTCGGATGAT	Will amplify 219-bp fragment of rat AhRNT (NM_012780.1)
	DS	CCGTTCCCCTCAAGGACTTC	
CAR	US	CTCTCCTGACAGGCCTGGGGT	Will amplify 156-bp fragment of rat CAR (NM_022941.3)
	DS	CGAAGCTCAGCTAGCAGGCCC	
CYP1A1	US	TCCCTGGGGTCCTAGAGAACACTCT	Will amplify 109-bp fragment of rat CYP1A1 (NM_012540.2)
	DS	TGTGGCTGATGTGAAGGCTGGG	
hCYP1A2	US	CACACCAGCCATTACAACCCTGCC	Will amplify 1608-bp coding sequence of human CYP1A2 (NM_000761.3)
	DS	TCAGTTGATGGAGAAGCGCAGCC	
gCYP1A2	US	GTGGTCACTGGCATCCACACCA	Will amplify 460-bp fragment of genomic rat *Cyp1a2* (NC_005107.3)
	DS	AAGGGCAAGCCCCAGGGTCC	
rCYP1A2	US	CGCATTGGCTCCACACCCGT	Will amplify 412-bp fragment of rat CYP1A2 (NM_012541.3)
	DS	TCTCCTCGCTCTTCCTGGGGA	
CYP1B1	US	CAGCTTTTTGCCTGTCACCC	Will amplify 180-bp fragment of rat CYP1B1 (NM_012940.2)
	DS	ATGAAGCCGTCCTTGTCCAG	
CYP2B1	US	CGCATGGAGAAGGAGAAGTCGAACC	Will amplify 151-bp fragment of rat CYP2B1 (NM_001134844.1)
	DS	CGACATGGGGGTACTTGAGCATCAG	
CYP2B2	US	CGCCTGTTGGAGCTGTTCTA	Will amplify 151-bp fragment of rat CYP2B2 (NM_001198676.1)
	DS	ACTTCTCCTCTCTCATCCATGC	
CYP2B3	US	CCCTTCTCCATAGGAAAGCGTA	Will amplify 269-bp fragment of rat CYP2B3 (NM_173294.1)
	DS	CCAGCAGGTCTCCCAGAATC	
CYP2C11	US	CTGCCATGGATCCAGTCCTAGTCC	Will amplify 88-bp fragment of rat CYP2C11 (NM_019184.2)
	DS	TTCCCTCTCCCAAAGCTCTGTCTCC	
CYP2C12	US	TGTGAGCACTCCTGCATTTCAGG	Will amplify 317-bp fragment of rat CYP2C12 (NM_031572.1)
	DS	AGAGCAAAAGTGCAAATCTCAGCGT	
CYP2C6	US	CTGTGACCAACCAGCTAAAGTCCAG	Will amplify 82-bp fragment of rat CYP2C6 (XM_003748910.1)
	DS	CTCCATGCGGGCTAGGCCCT	
CYP3A1/23	US	TGGCCCAGTGGGGATTATGGGG	Will amplify 183-bp fragment of rat CYP3A1/23 (NM_013105.2)
	DS	GGGACAGGTTTGCCTTTCTCTTGCC	
CYP3A2	US	TGGCAAGGTC-GTGATGGAAC	Will amplify 72-bp fragment of rat CYP3A2 (NM_153312.2)
	DS	ACCAGATGTGGATGGAGATGG	
CYP3A18	US	GGAGGCCTGAACTGCTGAAGGAG	Will amplify 166-bp fragment of rat CYP3A18 (NM_145782.1)
	DS	AAGGCACAGGTTTGGGTCCAGGA	
CYP3A19	US	GCCCTGAAAGGTTCAGCAAG	Will amplify 282-bp fragment of rat CYP3A19 (NM_147206.2)
	DS	AGGCCATTCTACATCAAGCTCC	
GAPDH	US	TGACATCAAGAAGGTGGTGAAG	Will amplify 243bp of rat glyceraldehyde 3 phosphate dehydrogenase (NM_017008.3)
	DS	TTGTCATACCAGGAAATGAGCT	
GSTA2	US	GCACAGACCAGAGCCATTCT	Will amplify 508-bp fragment of rat GSTA2 (NM_017013.4)
	DS	GCAAAACATAAAGAAATTGGACAGT	
GSTA3	US	CACCGAGAACTCTTGATGTGT	Will amplify 256-bp fragment of rat GSTA3 (NM_0.1509.2)
	DS	CAATCTCCACCATGGGCACT	
GSTA4	US	CTGCTTTTTGGCCAAGTCCC	Will amplify 236-bp fragment of rat GSTA4 (NM_001106840.1)
	DS	GCCCTCTTCACTGCTAAAGCTA	
GSTA5	US	AAGACCGCCTTGGCAAAAGA	Will amplify 356-bp fragment of rat GSTA5 (NM_001010921.1)
	DS	AACATCAGAGCCTGGATTACAAG	
GSTK1	US	AAGCAGCTCTTCCAGGTTCC	Will amplify 458-bp fragment of at rat GSTK1 (NM_181371.2)
	DS	AGTCTGGCATTCAGGGTTGG	
GSTM1	US	AGACAGAGGAGGAGCGGATT	Will amplify 417-bp fragment of rat GSTM1 (NM_017014.1)
	DS	CTGTGAGTGCCAGTGTAGCA	
GSTM2	US	AAGCACAACCTTTGTGGGGA	Will amplify 377-bp fragment of rat GSTM2 (NM_177426.1)
	DS	ATTGGCTTGGAGAGGAAGCG	
GSTM3	US	GCGGACTTACTCACCCCATC	Will amplify 328-bp fragment of rat GSTM3 (NM_020540.1)
	DS	AAGTCAGGACTGCAGCAAACT	
GSTM4	US	TACTCACACCGGAGGCTAGT	Will amplify 498-bp fragment of rat GSTM4 (NM_001024304.1)
	DS	TTCACCAACGAACCACGTCT	
GSTM5	US	TCATGCCATCCGTATGCTCC	Will amplify 309-bp fragment of rat GSTM5 (NM_172038.1)
	DS	TTGTAGCAGAGCCGAACCAG	
GSTM6	US	GCAGCTCCGGAACTACTCTC	Will amplify 498-bp fragment of rat GSTM6 (NM_001109192.1)
	DS	GCCCTTCAAGAACTCAGGCT	
GSTO1	US	GCGAGTACCTGGATGAAGCA	Will amplify 242-bp fragment of rat GSTO1 (NM_001007602.1)
	DS	GAGCGAATTCCCACCGAAGA	
GSTO2	US	GTAGGATGTGAGACCAGCGG	Will amplify 327-bp fragment of rat GSTO2 (NM_001012071.1)
	DS	AGCACTCTGGTGTTGATGGG	
GSTP1	US	ACGCAGCTTTGAGTCCACAC	Will amplify 412-bp fragment of rat GSTP1 (NM_012577.2)
	DS	CAGGCAGGGCCTTCACATAG	
GSTT1	US	CGTGCTCGTGTGGATGAGTA	Will amplify 399-bp fragment of at rat GSTT1 (NM_053293.2)
	DS	GTCAGCAGGTGGACAGTCTC	
GSTT2	US	TTTCAGTTGCGTACCGTGGA	Will amplify 250-bp fragment of rat GSTT2 (NM_012796.2)
	DS	CAAAGGTGCCACGGATGTTG	
GSTT3	US	TTTGCCCAGGTGAACCCTTT	Will amplify 496-bp fragment of rat GSTT3 (NM_001137643.1)
	DS	CCTCACCTCTTCACTTGCGT	
GSTT4	US	GATCACGGGTGAGGAGGTTC	Will amplify 228-bp fragment of rat GSTT4 (NM_001109675.1)
	DS	TCCACCCGCATTCTCCATTC	
GSTZ1	US	AGGAGGGAACGCCATCTAGT	Will amplify 238-bp fragment of rat GSTZ1 (NM_001109445.1)
	DS	TGTTGCCCGCCATCCTTTAT	
MGST1	US	ACGAGGTGTTGATGGCCTTT	Will amplify 354-bp fragment of rat MGST1 (NM_134349.3)
	DS	GCTGAGGAAGGGGAGTCAAG	
MGST2	US	TTTGCTTTGCAAGTCGGACG	Will amplify 236-bp fragment of rat MGST2 (NM_001106430.1)
	DS	GCTTCTGCATAGCCCCAGAA	
PAPSS1	US	CTCTCTTACCACTCGGCCTC	Will amplify 313-bp fragment of rat PAPSS1 (NM_001106471.1)
	DS	AAGTGTAGCACGGAATGCCA	
PAPSS2	US	CCGTGTTACTCCCTGGATGG	Will amplify 600-bp fragment of rat PAPSS2 (NM_001106375.2)
	DS	AAAGCCTTTGAGCGGAGTGG	
PXR	US	GCTCCTGCTGGACCCGTTGA	Will amplify 115-bp fragment of rat PXR (NM_052980.2)
	DS	GCCAGGGCGATCTGGGGAGAA	
RXRα	US	TCTTCATCCCTGAGCTCTCCA	Will amplify 263-bp fragment of rat RXRα (NM_012805.2)
	DS	TTCATGGGTGAGTTGAGCTGG	
RXRβ	US	GACAGCTCCTCCCCAAATCC	Will amplify 213-bp fragment of rat RXRβ (NM_206849.3)
	DS	GGAGTTAATCTGAGGGCTGC	
SULT1A1	US	ACACATCTGCCCCTGTCCT	Will amplify 77-bp fragment of rat SULT1A1 (NM_031834.1)
	DS	GCATTTCGGGCAATGTAGA	
SULT1B1	US	CGAGATGTTATTACCTCTAAAGTTCCA	Will amplify 88-bp fragment of rat SULT1B1 (NM_025513.1)
	DS	GAGTTTTCTTCAAGAGTTCAACACC	
SULT1C2	US	TCTGCCCTTGAGGTATCCAG	Will amplify 90-bp fragment of rat SULT1C2 (NM_133547.4)
	DS	GCGGCTGTAATCTGCTCAA	
SULT1C2A	US	TCTGCCCTTGAGGTATCCAG	Will amplify 87-bp fragment of rat SULT1C2A (NM_001013177.2)
	DS	CAGGGAAGAAGGTTTAGTTCCA	
SULT1C3	US	GGTACCCTGGGAGAATACATTG	Will amplify 84-bp fragment of rat SULT1C3 (NM_031732.2)
	DS	CCACCATCCCTTTACATGGT	
SULT1D1	US	CCTCGACTGGTGAAGACACA	Will amplify 87-bp fragment of rat SULT1D1 (NM_021769.1)
	DS	CCGTGCCACATAAATCATCTT	
SULT1E1	US	GAGAAATTTATGGAAGGGCAAG	Will amplify 103-bp fragment of rat SULT1E1 (NM_012883.1)
	DS	CATAGAACATAAACAAAACACGTGAA	
SULT2A1	US	TGGGGTAATTCAACTCTTGTGA	Will amplify 102-bp fragment of at rat SULT2A1 (NM_131903.1)
	DS	GATGTGCTCAAACCATGATCC	
SULT2A2	US	TCTTCAGTTCCAAGGCCAAG	Will amplify 118-bp fragment of rat SULT2A2 (NM_001025131.1)
	DS	GTTCCCAGCGAGTCTGGTT	
SULT2A6	US	AAGACAACTCTTGCGAAGAAGC	Will amplify 96-bp fragment of rat SULT2A6 (NM_012695.3)
	DS	GATGTGCTCAAACCATGATCC	
SULT2B1	US	GGTGATTTACTTGGGCCGGA	Will amplify 420-bp fragment of rat SULT2B1 (NM_001039665.1)
	DS	CAGTCGCCACTGATCCCTTT	
SULT4A1	US	CGGAAGTTGCTTGGAAACAG	Will amplify 60-bp fragment of rat SULT4A1 (NM_031641.1)
	DS	CATCTCACTCCTCGGCTCTC	
SULT5A1	US	CTCCAGAAGGACCTAACTTTGC	Will amplify 69-bp fragment of rat SULT5A1 (NM_001106194.1)
	DS	AATGGTTGAGCGAGGTTCC	
SULT6B1	US	TCCGAGCTTTGGATGCCTTT	Will amplify 608-bp fragment of rat SULT6B1 (NM_001192017.1)
	DS	CTGGGATTTTGCTCGCATCG	
UGT1A1	US	TGGCCTCTCTGGAACAAAGC	Will amplify 486-bp fragment of rat UGT1A1 (NM_012683.2)
	DS	CTCCGGAGGCGTTGACATAG	
UGT1A3	US	TATGGCTCTCTGGCGAGACT	Will amplify 347-bp fragment of rat UGT1A3 (NM_201424.2)
	DS	GGTCTAGTTCCGGTGTAGCG	
UGT1A5	US	GACTCCATGTGACCCTGCAA	Will amplify 461-bp fragment of rat UGT1A5 (NM_001039549.1)
	DS	CCCACACGGAATCACAGGAT	
UGT1A8	US	AGAGGTGAGTTGGCACATGG	Will amplify 343-bp fragment of rat UGT1A8 (NM_175846.2)
	DS	TGGCAAAATATTCCCCCGCT	
UGT1A9	US	CCATCAATAATTTTTGCCAAAGACA	Will amplify 393-bp fragment of rat UGT1A9 (NM_201425.2)
	DS	GGAGGCGTTGACATAGGCTT	
UGT2B1	US	GCAAAGCACTCATTTGGAACAAG	Will amplify 415-bp fragment of rat UGT2B1 (NM_173295.1)
	DS	TCCAAGTCCCAGAAGGTTCG	
UGT2B2	US	TCGACTTTTGGTTCGAGAGACTT	Will amplify 317-bp fragment of rat UGT2B2 (NM_031533.5)
	DS	TGCAATTGCGTTGGCCTTTT	
UGT2B3	US	CCTGCTACAGATAAGTTGCTGTTTC	Will amplify 380-bp fragment of rat UGT2B3 (NM_153314.2)
	DS	GCTGCTTGTTTGAAACGGTGT	

Abbreviation: RXR, retinoid X-receptor.

##### Western blotting.

Western blotting was performed essentially as previously described (Marek *et al.*, 2005). The antibodies used were goat anti-CYP2C11 and anti-CYP1A1 (Daiichi Chemical Co., Tokyo, Japan), and mouse anti-CYP3A1 and anti-CYP2B1 + 2B2 (Abcam, Cambridge, UK). Detection was carried out using the relevant anti-IgG horseradish peroxidase (HRP)–conjugated secondary antibody and ECL-based chemiluminescent detection (GE Healthcare, Amersham, UK).

##### Immunocytochemistry.

Immunofluorescence was carried out as previously described (Marek *et al.*, 2009) using rabbit anti-CYP2E1 (Abcam) or mouse anti-bromodeoxyuridine (BrdU) (BD Biosciences, Oxford, UK) and a fluorescein isothiocyanate– or HRP-conjugated secondary antibody. Slides were imaged using a Leica brightfield (Milton-Keynes, UK) or Zeiss fluorescence microscope (Cambridge, UK).

##### Alkoxyresorufin dealkylase assays.

Ethoxyresorufin o deethylase (EROD), pentoxyresorufin o depentylase (PROD), and methoxyresorufine o demethylase (MROD) activities were determined in cell lysates using a temperature-controlled 96-well fluorimetric plate reader. Cell pellets were lysed in TM buffer (50mM Tris-HCl, 25mM MgCl_2_, pH 7.5) and 50 μl of the lysate was added to 100 μl of 4μM ethoxyresorufin, 20μM pentoxyresorufin, or 2μM of methoxyresorufin. Plates were prewarmed at 37°C for 10min and reactions were initiated by addition of 50 μl of 500μM NADPH. Resorufin generated was determined by monitoring fluorescence (excitation at 522nm, emission at 586nm) using authentic resorufin as standard. Fluorescence was measured every 1–2min at 37°C for 20min with activities normalized to total cell protein.

##### Luciferin-IPA assay.

Luciferin-IPA (Promega, Southampton, UK) was used to determine CYP3A-mediated activities in cell lysates following the manufacturer’s instructions, except that NADPH was added to give a final concentration of 1.5mM. Results were normalized to total cell protein.

##### Comet assay.

DNA damage was assessed by comet assays essentially as outlined (Jowsey *et al.*, 2009). DNA damage, expressed as the mean olive tail moment, was calculated from 50 individual cells using AutoComet software (Tritek Corp., Summerduck, Virginia). When required, cells were pretreated daily for 3 days with inducers or vehicle with addition of potentially genotoxic compounds on day 3. After a further 24h, cells were harvested and analyzed. Benzo[α]pyrene and PhIP were added from 100-fold molar concentrated stocks in dimethysulfoxide (DMSO), therefore their respective vehicle control was 1% (vol/vol) DMSO. To control for any effects on cell viability and proliferation (which can give false positive results), compounds were separately tested for cytotoxicity or DNA synthesis. Cytotoxicity was assessed by embedding cells in low melting point agarose, followed by lysis and a 3-min incubation in 0.4M Tris buffer pH 7.0 and staining with SYBR gold (Invitrogen) as described by Mosesso *et al.* (2012). DNA synthesis was assessed using BrdU incorporation into DNA with staining carried out essentially as described in Mosesso *et al.* (2012).

##### Transporter efflux assays.

Efflux transporter function was determined by loading cells with 5μM cholyl-lysyl-fluorescein (CLF) obtained from BD Biosciences, 1μM Hoechst 33342, or 1μM 5-chloromethylfluorescein diacetate (CMFDA) for 30min as per standard culture. Cells were then washed in PBS and incubated with a transporter inhibitor (100μM troglitazone, 5μM cyclosporine A, 1μM KO143, or 10μM MK571) or vehicles for 30min as per standard culture. After 3 washes in PBS, levels of fluorophore retained within the cells were determined using the following fluorimetric settings: CLF, excitation at 490nm, emission at 550nm; Hoechst 33342, excitation at 355nm, emission at 480nm; and CMFDA, excitation at 480nm, emission at 520nm.

##### Genomic sequencing of B-13 cells.

B-13 cells were expanded in ten 75-cm^2^ culture flasks and the medium removed (and confirmed negative for bacterial and mycoplasma contamination) prior to DNA isolation as previously outlined (Wallace *et al.*, 2010c). The DNA was then subjected to mate-pair library construction and SOLiD Whole Genome sequencing (Becton Coulter Genomics), sequencing at 30× coverage of a haploid rat genome. The SOLiD color space raw data were mapped to the reference Wistar rat genome sequence (RN4) using the Corona Lite platform (v 4.2.1) from Applied Biosystems. A maximum of 4 mismatches per 50-bp read were allowed in the mapping step. The pairing pipeline of Corona was used to correctly match mate pairs mapped to the reference (assuming a fragment size distribution of 260–2160bp, determined from a sample of the correctly matched reads). The single nucleotide polymorphism, small InDel, and large InDel pipelines used the results of these procedures to find variants in the mapped reads compared with the reference. These variants were then interrogated using the Integrative Genomic Viewer software (Robinson *et al.*, 2011; Thorvaldsdóttir *et al.*, 2013) available at https://www.broadinstitute.org/igv/ and by alignment with the reference Wistar rat genome sequence RN4.

##### hCYP1A2 cloning.

The full-length coding sequence of the human *CYP1A2* gene (hCYP1A2) was amplified from cDNA prepared from donor liver samples. Human liver tissue was obtained with informed consent from patients undergoing surgical liver resections at the Freeman Hospital, Newcastle, with ethical approval from the Newcastle and North Tyneside 1 Ethics Research Committee (NRES—The Newcastle Hepatopancreatobiliary Research Tissue Bank—10/H0906/41). PCR products were separated by agarose gel electrophoresis and extracted using a gel extraction kit (Qiagen, Manchester, UK). The extracted DNA was cloned into a pENTR/D-TOPO vector (Invitrogen) and transformed into TOP10 competent cells (Invitrogen) following the manufacturer’s protocol. Plasmid DNA was purified using Spin minipreps kits (Qiagen) and plasmid containing the CYP1A2*1 (wild-type hCYP1A2) cDNA transferred to a pT-Rex-DEST30 vector (Invitrogen) using LR Clonase II (Invitrogen) following the recommended protocol prior to transfection into B-13^−TR^ cells using effectene as previously described (Wallace *et al.*, 2010c). Stable transfectants were selected using geneticin and blastocidin (both Invitrogen) at 400 and 8 μg/ml, respectively, for 2 weeks to generate cell lines expressing both the tetracycline repressor and hCYP1A2 (B-13^−TR/h1A2^). B-13^−TR/h1A2^ were routinely maintained in culture medium supplemented with 250 μg/ml geneticin and 4 μg/ml blastocidin.

##### Statistics.

The student’s 2-tailed *t* test was used to determine significant difference between groups. Significance was achieved where *p* < .05. For comet assays, statistical significance was tested using ANOVA and significance between groups tested using the Bonferroni-Holm *ad hoc* test.

## RESULTS

### 

#### B-13/H Cells Express an Hepatic Phenotype and Male Pattern of CYP Expression

**FIG. 1.**
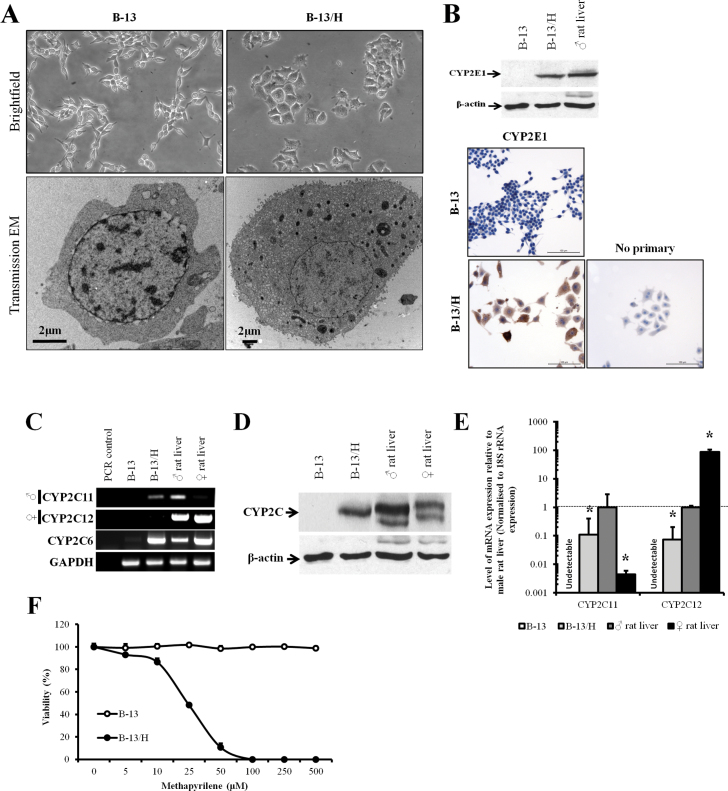
B-13 cells differentiate to hepatocyte-like B-13/H cells and express a male pattern of CYP. A, Representative brightfield (×100) and electron photomicrographs (×3400) of B-13 and B-13/H cells. B, Western blot (upper panels) and immunocytochemical staining for CYP2E1 in B-13 and B-13/H cells. For the Western blot, each lane contains 20 µg cell protein/lane. Data typical of at least 50 separate experiments. C, RT-PCR for the indicated transcripts, ♂, male-predominant form, ♀, female-predominant form. Data typical of at least 5 separate experiments. D, Western blot for CYP2C11 in the indicated extracts. Each lane contains 20 µg protein/lane. Data typical of at least 4 separate experiments. E, Quantitative RT-PCR for CYP2C11 and CYP2C12; * indicates statistical significantly different from male rat liver. Data are the mean and SD of 3 separate determinations from the same experiment, typical of at least 3 separate experiments. F, Toxicity of methapyrilene in B-13 and B-13/H cells after treatment for 24h with viability assessed by trypan blue exclusion. Data are the mean and SD of 3 separate samples from the same experiment, typical of 3 separate experiments. Abbreviations: CYP, cytochrome P450; RT-PCR, reverse transcriptase-PCR.

Figure 1A demonstrates that the B-13 cells used in these studies formed hepatocyte-like B-13/H cells after treatment with 10nM DEX for 14 days. The alteration from B-13 to B-13/H phenotype was characterized by a reduction in proliferation and an increase in cell size as previously observed (Wallace *et al.*, 2010b). Ultrastructural analysis by transmission electron microscopy demonstrated that there was an increase in the cytoplasmic ratio and a significant increase in the number of mitochondria (Fig. 1A) from 5±2 to 38±20 mitochondrion/cell in randomly selected B-13 and B-13/H cells, respectively. Figure 1B further shows that there were rat hepatocyte levels of CYP2E1 expression in B-13/H cells induced from undetectable levels in B-13 cells. CYP2E1 was present in 95%–100% of B-13/H cells as determined by immunohistochemical analysis (Fig. 1B). These data are typical of the response of B-13 cells to exposure to 10nM DEX and these endpoints were used throughout the following studies to confirm differentiation to B-13/H cells.

Cytogenetic and fluorescent *in situ* hybridization studies have established that B-13 cells are rat cells and were derived from a male rat because all cells contain Y chromosomes (Fairhall *et al.*, 2013a). Because several rodent hepatic CYPs are expressed in a sexually dimorphic pattern (Morgan *et al.*, 1985; Waxman and Holloway, 2009), the expression of the major hepatic CYP forms was therefore examined. Figure 1C indicates that B-13 cells did not express detectable levels of the major hepatic male or female CYP2C11 and CYP2C12 mRNA transcripts, respectively, whereas in the B-13/H phenotype, the CYP2C11 mRNA transcript was expressed, but not the CYP2C12.

Quantitative RT-PCR suggests that B-13/H cells expressed approximately 10% of the CYP2C11 mRNA levels found in adult male rat liver and 0.1% of the CYP2C12 mRNA levels found in female rat liver (Fig. 1D). CYP2C6 is a related CYP2C form that does not show a sexually dimorphic pattern of expression (Morgan *et al.*, 1985; Waxman and Holloway, 2009). Figure 1C shows that this gene was expressed in B-13/H cells from undetectable levels in B-13 cells. Quantitative RT-PCR indicated CYP2C6 mRNA was induced > 300-fold in B-13/H cells (data not shown). Western blotting for CYP2C proteins essentially supports the transcript data. Figure 1D indicates that there was no detectable CYP2C protein in B-13 cells, whereas B-13/H cells expressed immunodetectable protein at similar levels to those present in male rat liver. To establish whether the CYP2C11 expressed in B-13/H cells was functional, B-13 and B-13/H cells were treated with methapyrilene, which has been shown to be activated to a toxic product by CYP2C11 (Ratra *et al.*, 1998). Figure 1F shows that the viability of B-13 cells was unaffected by methapyrilene exposure at concentrations of up to at least 500µM, whereas methapyrilene treatment caused a dose-dependent loss of viability in B-13/H cells, with an EC_50%_ concentration of approximately 20µM.

#### The Aryl Hydrocarbon Receptor Activator β-NF Induces CYP1A1 Expression in B-13 and B-13/H Cells and Sensitizes Cells to the Genotoxic Effects of Benzo[α]pyrene

**FIG. 2.**
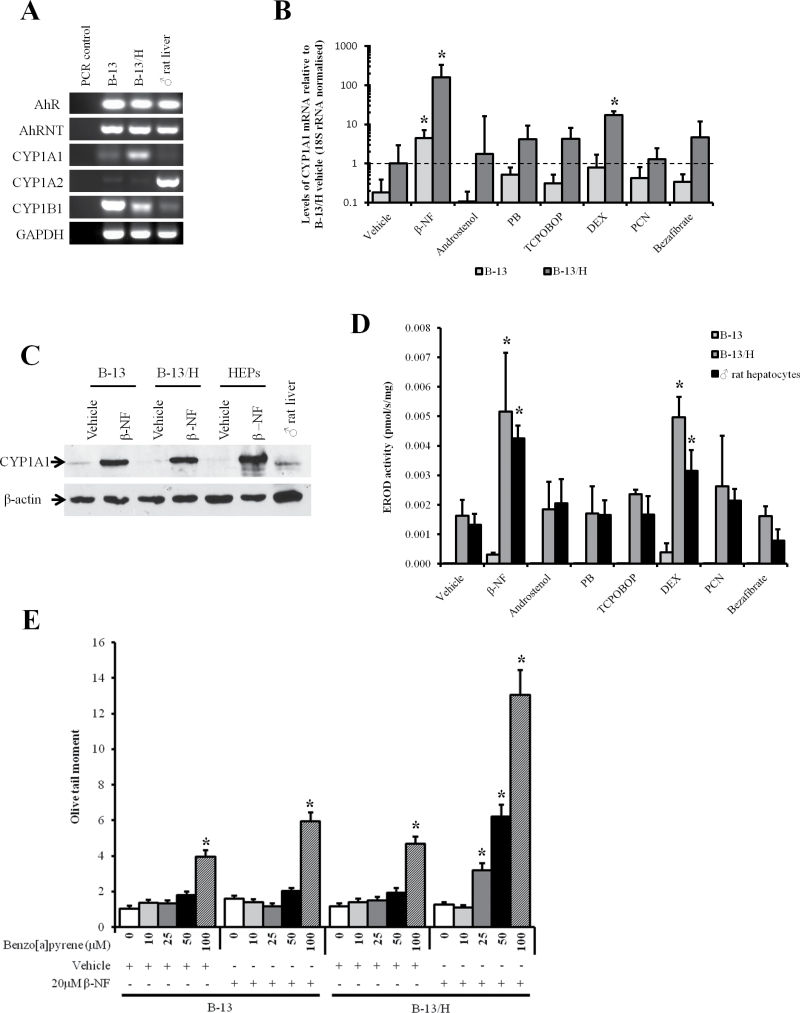
B-13/H cells express the AhR, induce functional CYP1A1 in response to challenge with an AhR agonist, and activate benzo[α]pyrene to a DNA-damaging species. A, RT-PCR for AhR and CYP1-related transcripts. Results typical of at least 5 separate experiments. B, Quantitative RT-PCR for the CYP1A1 transcript in B-13 and B-13/H cells after treatment with inducers as outlined in Materials and Methods section. Data are the mean and SD of 3 separate samples from the same experiment, typical of 3 separate experiments. *Statistically different from the same cell type treated with vehicle alone. C, Western blot for CYP1A1 following vehicle or β-NF treatment as outlined in Materials and Methods section, each lane contains 20 µg cell protein/lane. Data are typical of at least 3 separate experiments. D, EROD activity in B-13, B-13/H, and hepatocytes following the treatment with inducers as outlined in Materials and Methods section. Data are the mean and SD of 3 separate samples from the same experiment, typical of 3 separate experiments. *Statistically different from the same cell type treated with vehicle alone. E, Comet assay (olive tail moment) in response to benzo[α]pyrene exposure in B-13 and B-13/H cells after pretreatment with vehicle or the CYP1A1 inducer β-NF. *Significantly different from vehicle-only-treated cells. Abbreviations: AhR, aryl hydrocarbon receptor; AhRNT, AhR nuclear translocator; β-NF, β-naphthoflavone; CYP, cytochrome P450; DEX, dexamethasone; EROD, ethoxyresorufin o deethylase; PB, phenobarbital; PCN, 5-pregnen-3β-ol-20-one-16α-carbonitrile; RT-PCR, reverse transcriptase-PCR; TCPOBOP, 1,4-Bis-[2-(3,5-dichloropyridyloxy)]benzene, 3,3′,5,5′-Tetrachloro-1,4-bis(pyridyloxy)benzene.

The CYP1A subfamily is of toxicological significance due to its metabolism of a range of carcinogens such as polyaromatic hydrocarbons (CYP1A1) and heterocyclic amines (CYP1A2) (Conney, 1982; Ma and Lu, 2007). These genes are inducible through their regulation by the aryl hydrocarbon receptor (AhR) (Nebert *et al.*, 2004). Figure 2A shows that both B-13 and B-13/H cells expressed the AhR and its heterodimeric partner AhR nuclear translocator mRNA transcripts. The cells also expressed CYP1A1 mRNA although CYP1A2 was not detectable in either B-13 or B-13/H cells. Interestingly, the related CYP1B1 mRNA transcript—often expressed at high levels in tumor cells (McFadyen and Murray, 2005)—was expressed in B-13 cells but downregulated to near hepatocyte levels in B-13/H cells (Fig. 2A). Quantitative RT-PCR confirmed that CYP1A1 mRNA expression was approximately 10-fold higher in B-13/H cells versus B-13 cells. Both B-13 and B-13/H cells induced their levels of CYP1A1 mRNA levels significantly by approximately 100-fold in response to exposure to β-NF (Fig. 2B). In contrast, nuclear receptor activators that regulate induction of other major hepatic CYP subfamilies (andostenol, PB, and TCPOBOP activate the constitutive androstane receptor [CAR; Qatanani and Moore, 2005]; DEX [at 10µM] and PCN activate the pregnane X-receptor [PXR; Kliewer *et al.*, 1999]; bezafibrate activates the peroxisome proliferator-activated receptor α [Kliewer *et al.*, 1999]) had no significant effect on CYP1A1 mRNA levels, with the exception of DEX at 10µM (although a similar response was seen in rat hepatocytes treated with this high concentration of DEX; Supplementary Figure 1).

Figure 2C indicates that the levels of CYP1A1 protein were relatively low in both untreated B-13 and B-13/H cells but that β-NF treatment induced a similar level of expression of CYP1A1 to that seen in β-NF-treated rat hepatocytes. These data were confirmed at the level of enzyme activity using EROD as a CYP1A1 probe substrate (Fig. 2D). The specific levels of EROD activity after treatment with β-NF as well as other nuclear receptor activators in B-13/H cells in particular showed both a quantitative and qualitative similarity to activities observed with rat hepatocytes. Although the fold induction in EROD activity after treatment with β-NF was relatively low (maximum 2.5-fold) compared with both fold changes at the level of mRNA and protein, this was likely due to metabolism of the substrate by other enzymes in noninduced cells because rat hepatocytes give similar levels of activity in both untreated and β-NF-treated cells. This conclusion is supported by the observation that the CYP1A1-activated procarcinogen benzo[α]pyrene did not give rise to detectable levels of DNA damage in noninduced B-13 or B-13/H cells (Fig. 2E). However, in both B-13 and B-13/H cells, benzo[α]pyrene treatment resulted in a dose-dependent increase in DNA damage (detectable at 100µM, the highest concentration examined), with β-NF pretreatment resulting in increased sensitivity in B-13/H cells (DNA damage detectable by 25µM). This latter observation of increased sensitivity to benzo[α]pyrene-dependent DNA damage by B-13/H cells was likely due to the more robust induction of EROD activity compared with B-13 cells (Fig. 2D). In all cases, the increase in the appearance of comets with benzo[α]pyrene treatment in B-13/H was associated with direct damage to the DNA. At the concentration range of benzo[α]pyrene employed, comets were not associated with cytotoxicity or with a significant increase in DNA synthesis (Supplementary Figure 2).

#### Stable Transfection of Functional Human CYP1A2 into B-13 Cells Sensitizes B-13/H Cells to the DNA-Damaging Effects of the Cooked Food Mutagen PhIP

**FIG. 3.**
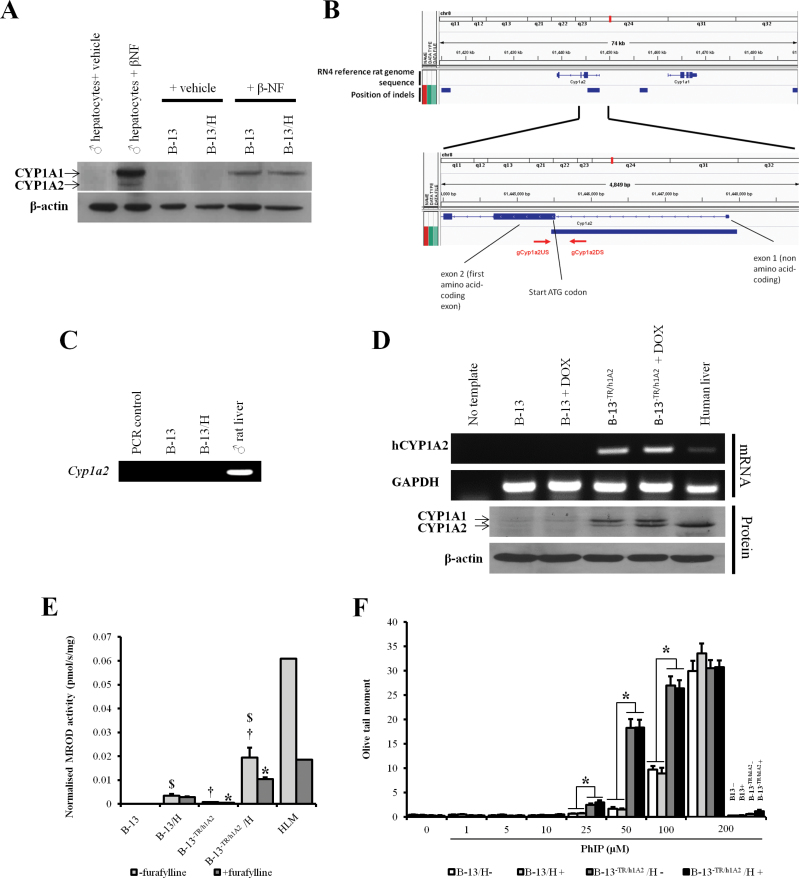
B-13^−TR/h1A2^ (humanized) cells express functional hCYP1A2, which activates PhIP to a DNA-damaging species. A, Western blot for CYP1A1 and CYP1A2 after treatment with β-NF inducer as indicated. Each lane contains 20 µg cell protein/lane. B, Schematic diagram of B-13 CYP1A2 genomic region indicating deletion and position of genomic primer hybridization used in PCR amplification. C, PCR using genomic DNA from the indicated source and gCyp1a2US and gCyp1a2DS primers. D, RT-PCR for human CYP1A2 (upper panels) and Western blot (lower panels) for CYP1A1 and CYP1A2 in B-13 and B-13^−TR/h1A2^ with or without doxycycline treatment (2 μg/ml). Data typical of 3 separate experiments. E, MROD assay in B-13 and B-13^−TR/h1A2^ with or without addition of furafylline (100μM). Data are mean and SD of 3 separate determinations from the same experiment, typical of 3 separate experiments; *statistically significantly activity compared with extract without addition of furafylline, ^$^statistically significant different activity compared with respective B-13 equivalent, ^†^statistically significant different activity compared with parent B-13 cells. F, Comet assay (olive tail moment) with B-13 or B-13^−TR/h1A2^ in response to PhIP exposure with or without doxycycline treatment. * indicates significantly different. Abbreviations: β-NF, β-naphthoflavone; CYP, cytochrome P450; HLM, human liver microsomes; MROD, methoxyresorufine o demethylase; PhIP, 2-amino-1-methyl-6-phenylimidazo(4,5-b)pyridine; RT-PCR, reverse transcriptase-PCR.

Because both B-13 and B-13/H cells failed to express any detectable CYP1A2 mRNA (Fig. 2A), proteins from B-13 and B-13/H cells were examined by Western blotting under chromatographic conditions that separate the closely related CYP1A1 and CYP1A2 proteins. Figure 3A suggests that although B-13 cells, B-13/H cells, and rat hepatocytes induced readily detectable levels of CYP1A1 protein in response to treatment with β-NF, only rat hepatocytes induced expression of CYP1A2 protein.

To establish whether the *Cyp1a2* genes are disrupted in B-13 cells, B-13 DNA was sequenced as outlined in the Materials and Methods section and aligned to the reference rat genome sequence. This analysis indicated that the B-13 genome was disrupted at the 5′ end of the *Cyp1a2* gene such that the proximal promoter, first (noncoding) exon, first intron, and part of the coding second exon (including the translation start site) were deleted (Fig. 3B). To confirm that all B-13 *Cyp1a2* alleles were disrupted, the region around the predicted deletion region was analyzed by PCR. Figure 3C suggests that no functional *Cyp1a2* gene is present in the B-13 cells because primers designed to amplify a genomic region at the 3′ end of the predicted deleted region failed to amplify a fragment from DNA isolated from B-13 DNA. In contrast, a fragment of the predicted size was amplified when Wistar rat DNA was used as template.

The human CYP1A2 cDNA sequence was therefore cloned as outlined in the Materials and Methods section and stably transfected into B-13^−TR^ cells expressing the doxycycline-regulated transcription factor to form B-13^−TR/h1A2^ cells. Figure 3D shows that the human CYP1A2 mRNA and protein were expressed in B-13^−TR/h1A2^ cells but not in the parent B-13 cell line. Doxycyclin treatment increased the expression of human CYP1A2 in B-13^−TR/h1A2^ cells; however, full repression of the transgene in the absence of doxycycline was not apparent (Fig. 3D).

To determine whether the hCYP1A2 protein was metabolically functional, cells were examined for their ability to metabolize methoxyresorufin. The effect of furafylline was also examined because this is a specific inhibitor of human CYP1A2 (Sesardic *et al.*, 1990). Figure 3E demonstrates that the parent B-13 possessed undetectable levels of MROD activity and on differentiation to B-13/H cells, MROD activity was not inhibitable by furafylline. In contrast, B-13^−TR/h1A2^ cells expressed low but detectable (and furafylline-inhibitable) levels of MROD activity. Conversion of these cells to a B-13^−TR/h1A2^/H phenotype (by treatment with 10nM DEX for 14 days) resulted in high levels of furafylline-inhibitable MROD activity. The effects of doxycyclin on transgene expression were minimal likely because of a leaky high constitutive expression of the transgene.

Figure 3F shows that treating parent B-13 cells or B-13^−TR/h1A2^ cells with up to 200µM PhIP did not result in any appreciable DNA damage. However, addition of PhIP to B-13/H or B-13^−TR/h1A2^/H cells resulted in a dose-response increase in DNA damage, with B-13^−TR/h1A2^/H cells showing significantly greater damage at 25–100µM PhIP compared with B-13/H cells. Doxycyclin treatment had little effect on the level of DNA damage, due to leaky expression of the transgene in cells treated with doxycyclin. This increase in the appearance of comets with PhIP treatment in B-13/H and B-13^−TR/h1A2^/H cells was associated with direct damage to the DNA. At the concentration range employed, comets were not associated with cytotoxicity or with a significant increase in DNA synthesis (Supplementary Figure 2).

#### CAR Activators Induce CYP2B1 mRNA in B-13/H Cells

The CYP2B subfamily of CYPs were some of the first CYPs to be purified to homogeneity and shown to be markedly induced by PB pretreatment. Although it has been established that CAR mediates this induction, PB does not bind directly to the CAR. The mechanism has recently been determined to be associated with an interference in epidermal growth factor (EGF) signaling (Mutoh *et al.*, 2013).

**FIG. 4.**
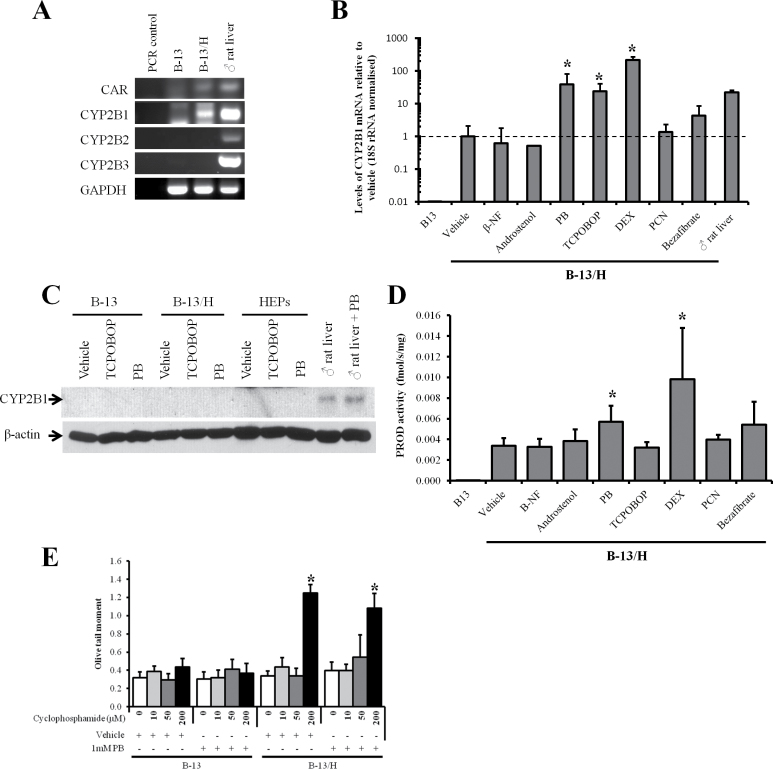
B-13/H cells express the CAR and robustly induce CYP2B1 mRNA in response to CAR activators, but expresses limited functional CYP2B1 protein. A, RT-PCR for the indicated transcripts. Data typical of 3 separate experiments. B, Quantitative RT-PCR for CYP2B1 mRNA in B-13 and B-13/H cells in response to indicated inducer treatments. Data are mean and SD of 3 separate determinations from the same experiment, typical of 3 separate experiments. *Statistically different from vehicle-only-treated cells. C, Western blot for CYP2B1 in B-13 cells, B-13/H cells, or hepatocytes treated with inducing concentrations of TCPOBOP or PB as outlined in Materials and Methods section, each lane contains 20 µg cell protein/lane. Data are typical of at least 3 separate experiments. D, PROD activity in B-13 cells and B-13/H cells treated with the indicated inducers. Data are mean and SD of 3 separate determinations from the same experiment, typical of 2 separate experiments. *Statistically different from the same cell type treated with vehicle alone. E, Comet assay (olive tail moments) in response to cyclophosphamide exposure in B-13 and B-13/H cells pretreated with or without PB. *Significantly different from vehicle-only-treated cells. Abbreviations: β-NF, β-naphthoflavone; CAR, constitutive androstane receptor; CYP, cytochrome P450; DEX, dexamethasone; PB, phenobarbital; PCN, 5-pregnen-3β-ol-20-one-16α-carbonitrile; PROD, pentoxyresorufin o depentylase; RT-PCR, reverse transcriptase-PCR; TCPOBOP, 1,4-Bis-[2-(3,5-dichloropyridyloxy)]benzene, 3,3′,5,5′-Tetrachloro-1,4-bis(pyridyloxy)benzene.

Figure 4A indicates that CAR mRNA was undetectable in B-13 cells, but detectable in B-13/H cells. Because only CYP2B1 mRNA was detectable in B-13/H cells, the expression of this CYP2B gene was examined after treatment with a variety of CYP inducers. B-13 cells did not respond to any inducer and CYP2B gene expression at the mRNA level was not detectable (data not shown). In contrast, Figure 4B demonstrates that the CAR activators PB and TCPOBOP induced at least a 24-fold increase in CYP2B1 mRNA expression. High concentrations of DEX also induced CYP2B1, likely through activation of the PXR (Lim and Huang, 2008). The mouse CARβ inverse agonist androstenol did not induce CYP2B1 mRNA (Fig. 4B). Supplementary Figure 3 indicates a qualitatively similar CYP2B1 mRNA induction response to CAR activators in primary hepatocytes; however, the relative levels of CYP2B1 mRNA remained below those present in untreated rat liver, whereas B-13/H cells induced their levels above untreated liver levels. Despite this robust induction of CYP2B1 mRNA in B-13/H after treatment with CAR activators, B-13/H cells did not express detectable levels of CYP2B protein (Fig. 4C) and there was a relatively low fold increase in CYP2B-mediated PROD activity in cell extracts (Fig. 4D).

Figure 4E shows that DNA damage caused by cyclophosphamide (which is metabolized by CYP2B to a genotoxic metabolite) was only apparent after treatment with 200µM cyclophosphamide in B-13/H cells, but not in B-13 cells. PB pretreatment did not significantly increase cyclophosphamide-dependent DNA damage in B-13/H cells, confirming that translation of CYP2B1 mRNA into functional protein limited functional CYP2B1 induction in B-13/H cells. The increase in the appearance of comets with cyclophosphamide treatment in B-13/H cells was however associated with direct damage to the DNA. At the concentration range employed, comets were not associated with cytotoxicity or with a significant increase in DNA synthesis (Supplementary Figure 2).

#### PXR Activators Induce a Quantitatively Liver Equivalent Induction of CYP3A1 in B-13/H Cells

The CYP3A subfamily is constitutively expressed and forms a major expressed subfamily in adult rat liver. The orthologous subfamily in man includes the major expressed gene *CYP3A4*, which is responsible for the metabolism of approximately 50% of drugs. Both CYP3A4 in man and CYP3A1 (also referred to as CYP3A23) in the rat are induced by activators of the PXR (Kliewer *et al.*, 2002).

**FIG. 5.**
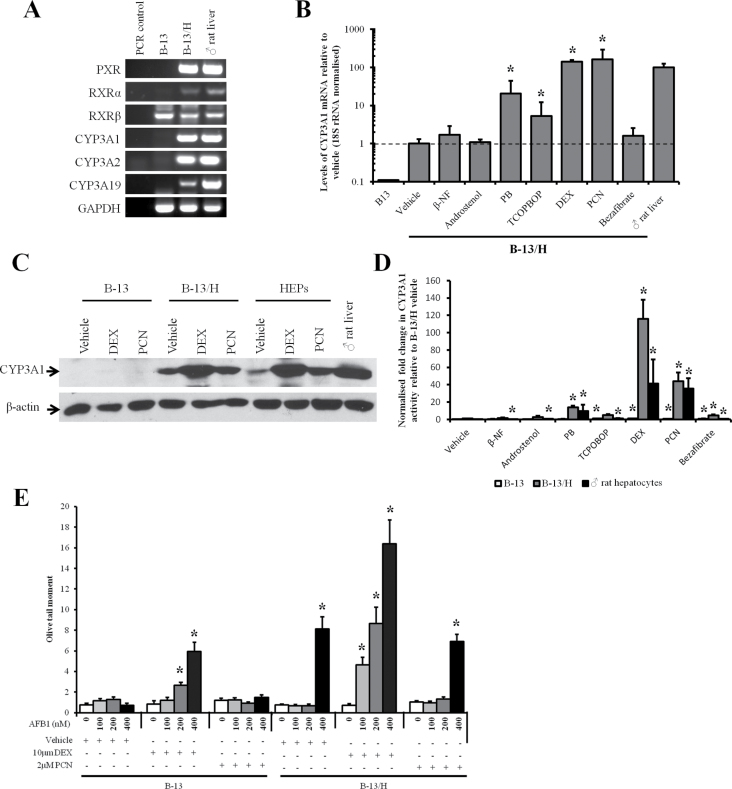
B-13/H cells express the PXR and robustly induce CYP3A1 mRNA, protein, and activity in response to PXR activators. A, RT-PCR for the indicated transcripts, data typical of 6 separate experiments. B, Quantitative RT-PCR for CYP3A1 mRNA in B-13 and B-13/H cells in response to indicated inducer treatments. Data are mean and SD of 3 separate determinations from the same experiment, typical of 3 separate experiments. *Significantly different from vehicle-only-treated cells. C, Western blot for CYP3A1 in B-13 cells, B-13/H cells, or hepatocytes treated with inducing concentrations of DEX or PCN as outlined in Materials and Methods section, each lane contains 20 µg cell protein/lane. Data typical of 3 separate experiments. D, CYP3A1 activity assessed by the luciferin-IPA assay in the indicated cell types following treatment with inducers as outlined in Materials and Methods section. Data are mean and SD of 3 separate determinations from the same experiment, typical of 3 separate experiments. *Significantly different from vehicle-only-treated cells. E, Comet assay (olive tail moment) in response to aflatoxin B1 exposure in B-13 and B-13/H cells pretreated with or without inducing concentrations of DEX or PCN. *Significantly different from vehicle-only-treated cells. Abbreviations: β-NF, β-naphthoflavone; CYP, cytochrome P450; DEX, dexamethasone; PB, phenobarbital; PCN, 5-pregnen-3β-ol-20-one-16α-carbonitrile; PXR, pregnane X-receptor; RT-PCR, reverse transcriptase-PCR; TCPOBOP, 1,4-Bis-[2-(3,5-dichloropyridyloxy)]benzene, 3,3′,5,5′-Tetrachloro-1,4-bis(pyridyloxy)benzene.

Figure 5A demonstrates that B-13/H cells—but not B-13 cells—expressed PXR mRNA as well as CYP3A1 and several other CYP3A transcripts. Figure 5B demonstrates that the PXR activators PB, DEX (at 10µM), and PCN induced up to approximately a 160-fold increase in CYP3A1 mRNA, to levels greater than found in intact adult rat liver. TCPOBOP also induced a lower fold increase in CYP3A1 mRNA (Fig. 5B) but did not give rise to an increase in CYP3A1 protein (data not shown). B-13 cells failed to induce detectable levels of CYP3A mRNAs after treatment with any inducer (data not shown).

A similar qualitative induction response to these inducers was also observed in primary hepatocytes (Supplementary Figure 4). When comparing with the levels of CYP3A1 protein induced by the most potent inducers—DEX (at 10µM) and PCN—similar levels of protein were expressed by B-13/H cells and primary hepatocytes (Fig. 5C). Despite significant increases in CYP3A1 mRNA over vehicle control, β-NF, PB, TCPOBOP, and bezafibrate failed to induce an increase in CYP3A1 protein (data not shown).

Figure 5D indicates that—in contrast to CYP2B induction—DEX (at 10µM) and PCN treatment resulted in the induction of functionally active CYP3A protein in B-13/H cells. To test this observation, B-13 and B-13/H cells were exposed to the procarcinogen aflatoxin B1, which is metabolized to a genotoxic product by CYP3A enzymes (Kelly *et al.*, 1997; Yamazaki *et al.*, 1995). Figure 5E shows that aflatoxin B1 did not cause DNA damage in B-13 cells or B-13 cells pretreated with PCN. In DEX inducer (ie, 10µM) pretreated B-13 cells, aflatoxin B1 exposure resulted in a dose-dependent increase in DNA damage, likely due to a glucocorticoid receptor–dependent promotion of B-13/H phenotype and subsequent induction of CYP3A1 expression within the short period of induction. Figure 5E also indicates that DNA damage was detectable in untreated B-13/H cells at 400nM aflatoxin B1. Pretreatment with DEX (at 10µM) resulted in a readily detectable dose-response increase in DNA damage, detectable at 100nM aflatoxin B1 (the lowest concentration examined). PCN pretreatment resulted in a less marked increase in DNA damage in response to aflatoxin B1 (Fig. 5E). This is likely due, in part, to a less robust induction of functional CYP3A (Fig. 5D). However, it is likely that other unknown factors may be involved.

The increase in DNA damage with aflatoxin B1 in B-13/H cells was likely associated with direct damage to the DNA. At the concentration range employed, comets were not associated with cytotoxicity or with a significant increase in DNA synthesis (Supplementary Figure 2).

#### Expression of Phase II Genes in B-13 Cells

**FIG. 6.**
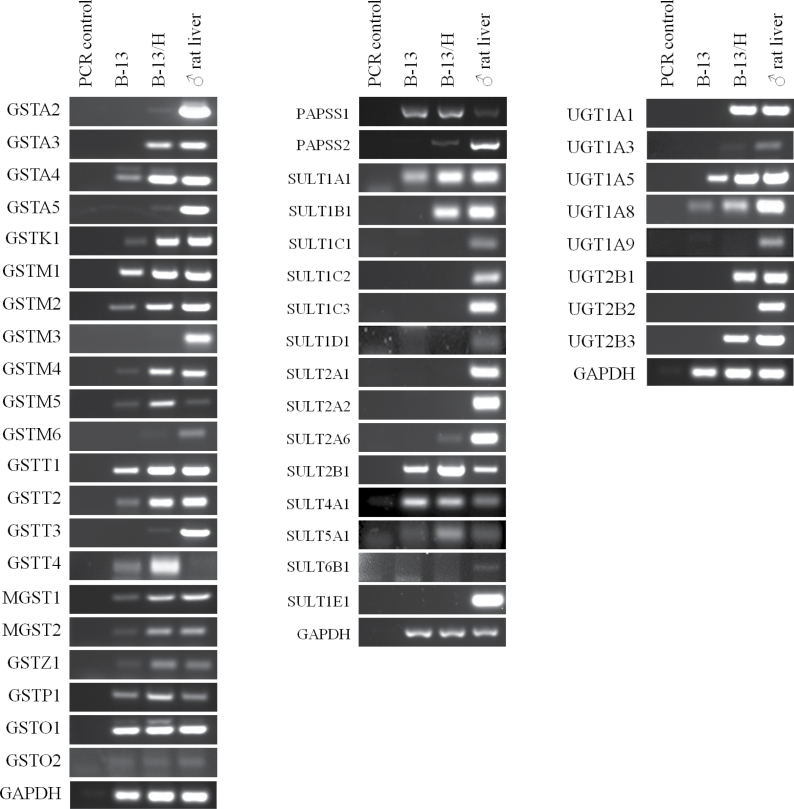
RT-PCR screens for phase II gene expression. RT-PCR for GST, SULT (and the generator of the cosubstrate PAPSS), and UDPGT transcripts. RT-PCR was performed as outlined in Materials and Methods section using primers as outlined in Table 1. Abbreviations: GST, glutathione S transferase; PAPSS, 3′-phosphoadenosine-5′phosphosulfate synthase; RT-PCR, reverse transcriptase-PCR; SULT, sulfotransferase; UDPGT, glucuronyltransferase.

Because the toxicity of many chemicals is affected by the activity of phase II enzyme systems, the expression of sulfotransferases (SULTs), glucuronyltransferases (UDPGTs), and glutathione S transferases (GSTs) was examined by RT-PCR. Figure 6 demonstrates that, in general, there is an upregulation in the expression of these genes in B-13/H compared with B-13 cells. B-13/H cells expressed 5 (from a total of 14) SULTs, 5 (from a total of 8 hepatic) UDPGTs, and 15 (from a total of 21) GSTs (Fig. 6).

#### B-13/H Cells Are Resistant to Proliferation In Vitro

**FIG. 7.**
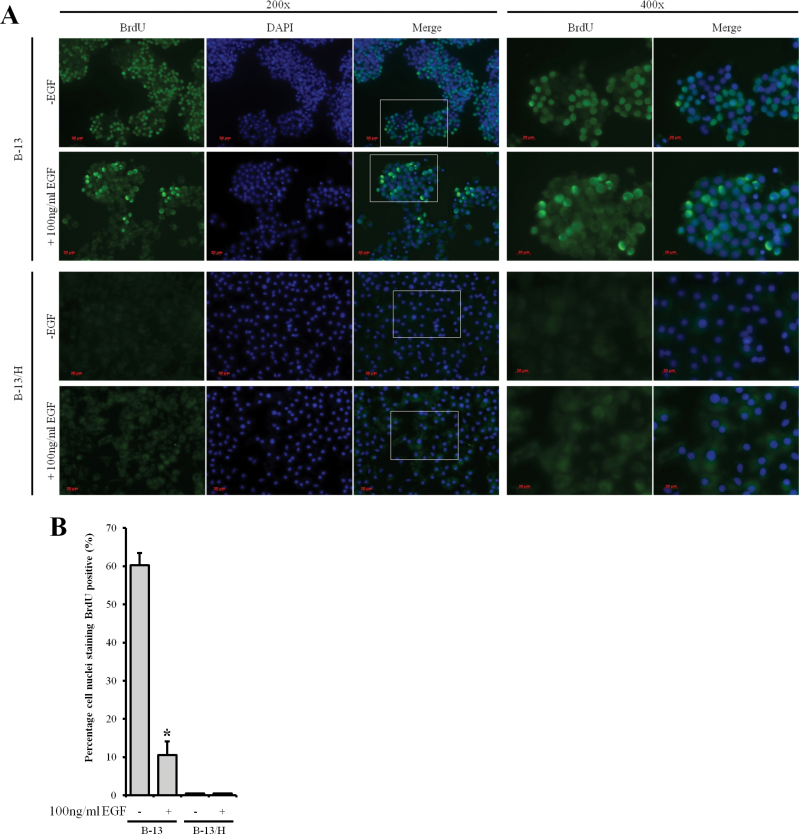
Effect of EGF treatment on DNA synthesis in B-13 and B-13/H cells. A, Immunofluorocytochemical staining for BrdU and DNA (DAPI) in B-13 and B-13/H cells. Where indicated, B-13 cells were treated with 100ng/ml EGF for 4 days. BrdU at 15µM was added to culture media 16h prior to fixation and analysis. B, Quantification of the percentage of cell nuclei (DAPI) positive for BrdU. Note, although B-13/H cells accumulated BrdU, this was not significantly accumulated into their nuclei. Data are the mean and SD of 3 separate experiments from the same experiment typical of at least 3 separate experiments. *Significantly different from vehicle-only-treated cells. Abbreviations: BrdU, bromodeoxyuridine; DAPI, 4′,6-diamidino-2-phenylindole; EGF, epidermal growth factor.

To establish whether B-13/H cells may be suitable for screening chemicals for clastogenicity, B-13 and B-13/H cells were examined for evidence of cell proliferation through incorporation of BrdU (because detection of clastogenicity is dependent on active mitosis in cells exposed to clastogenic chemicals). Note that genomic sequencing indicated that both the *K-ras* and *p53* genes were free of nonsynonymous mutations (Supplementary Figure 5A). The *K-ras* gene contains an insertion and a deletion in the proximal promoter and intronic region, respectively, which may account for relatively high levels of expression of K-ras in B-13 and B-13/H cells, in contrast to p53 levels that appear to be low (Supplementary Figures 5B and 5C). However, genomic sequencing suggests that the relatively high expression of K-ras will be of a normal *wild-type* protein product. Figure 7 demonstrates that BrdU is incorporated extensively into B-13 nuclei, with 60% showing evidence of active DNA synthesis. In contrast, B-13/H cells did not incorporate BrdU into their nuclei, suggesting that the cells enter replicative senescence under standard culture conditions. A number of growth factors have been examined for their ability to modulate B-13 cell behavior under serum-free conditions with EGF demonstrating the most potent activity in terms of B-13 proliferation and cell signaling activity (unpublished data). Figure 7 further shows, however, that EGF failed to stimulate B-13/H DNA synthesis (and similar results were seen with a variety of other hepatocyte mitogens—data not included). Interestingly, EGF treatment altered the phenotype of B-13 cells and potently stimulated DNA synthesis in a minority of cells, supporting our unpublished observations that B-13 cells are responsive to EGF. Despite this response in a limited number of cells, overall EGF reduced the proportion of B-13 cells actively undergoing DNA synthesis (Fig. 7B).

#### B-13 Cells Upregulate Transporter Expression and Are Functional in B-13/H Cells

**FIG. 8.**
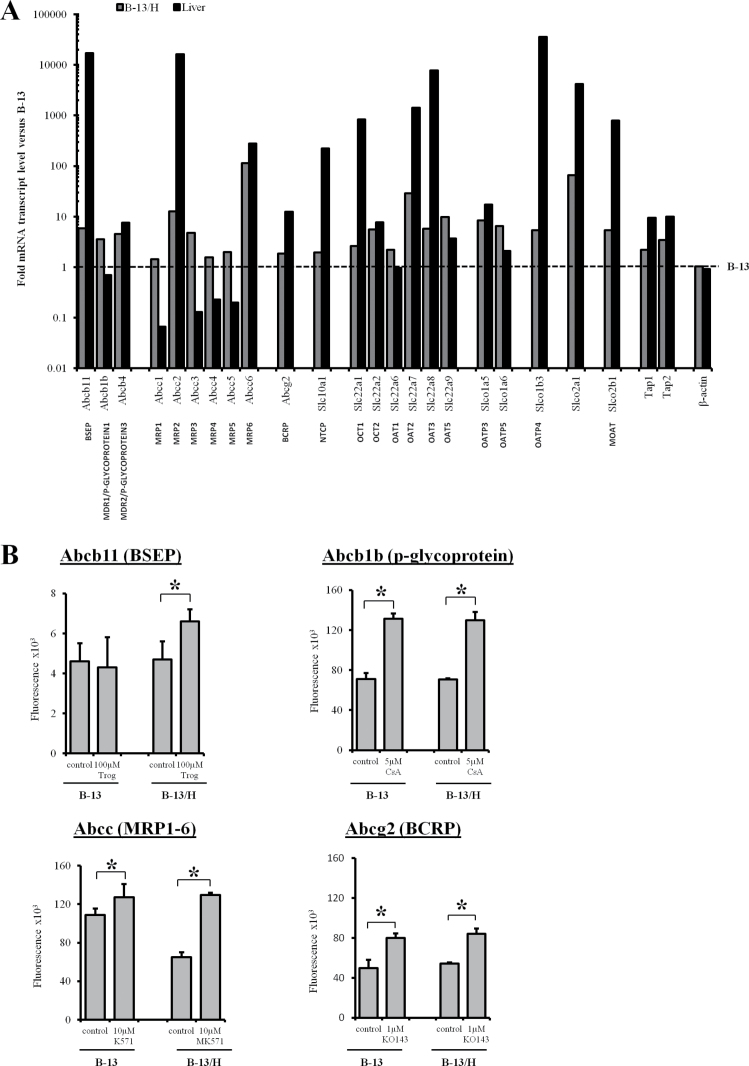
B-13 cells upregulate expression of functional transporter genes in B-13/H cells. A, Drug transporter expression in B-13, B-13/H, and control rat liver assessed by quantitative RT-PCR. Fold change in transcripts is expressed relative to B-13 cells after normalization to the housekeeping genes ribosomal protein large P1, hypoxanthine phosphoribosyltransferase 1, ribosomal protein L13A, lactate dehydrogenase A, and β-actin. B, Efflux assays for BSEP, p-glycoprotein, MRP1-6, and ABCG2 in B-13 and B-13/H cells, performed as described in Materials and Methods section. Data are mean and SD of 3 separate determinations from the same experiment, typical of 4 separate experiments. *Significantly different from control cells. Abbreviations: BCRP, breast cancer resistance protein; BSEP, bile salt export pump; MRP, multidrug resistance-associated protein; RT-PCR, reverse transcriptase-PCR.

Drug transporters are an important factor in determining drug and chemical toxicity and hepatocytes express a range of these transporters (Funk, 2008). A quantitative screen of transporters mRNA in B-13 cells, B-13/H cells, and rat liver showed that the differentiation of B-13 cells to B-13/H cells was associated with a general upregulation of all 23 transporter mRNAs, although many were expressed at levels lower than those seen in adult rat liver (Fig. 8A). Figure 8B shows that treatment of B-13/H cells with troglitazone, which inhibits bile salt exporter pump (BSEP) function (Funk *et al.*, 2001), caused a significant retention of CLF, indicating that the B-13/H cells expressed functional BSEP. Treatment of both B-13 and B-13/H cells with the p-glycoprotein inhibitor cyclosporine A (Nooter *et al.*, 1990) or breast cancer resistance protein (BCRP) inhibitor KO143 (Allen *et al.*, 2002) caused a significant retention of Hoechst 33342, which is a substrate for both p-glycoprotein and BRCP (Bunting *et al.*, 1999; Zhou *et al.*, 2001) (Fig. 8B). These data suggest that both B-13 and B-13/H cells express functional p-glycoprotein and BCRP. The multidrug resistance-associated protein (MRP) inhibitor MK57 (Leier *et al.*, 1994) caused a significant retention of CMFDA in B-13/H cells—but not B-13 cells—indicating that B-13/H cells expressed functional MRP transporters (Roelofsen *et al.*, 1997).

## DISCUSSION

The data in this paper support previous work showing that B-13 cells differentiate into hepatocyte-like B-13/H cells in response to glucocorticoid treatment (Marek *et al.*, 2003; Shen *et al.*, 2000; Wallace *et al.*, 2010c). The data extend these observations (for summary, see Table 2) to show that a male pattern of CYP is expressed in B-13/H cells and that the regulation of the major subfamilies of xenobiotic-metabolizing CYPs remains essentially intact. Furthermore, this paper demonstrates for the first time that transporter gene expression is increased in B-13 cells when they are converted to B-13/H cells, such that functional transport activity is detectable.

**TABLE 2:** Overview of B-13/H Cell Utility in Toxicity and Genotoxicity Studies

	Cell Phenotype		Comment
	B-13	B-13/H	
Constitutive hepatic CYP450 expression and associated activity			
CYP2E1	−	+	
CYP2C6	−	+	
CYP2C11	−	+	Male-specific isoform
CYP2C12	−	+ (low)	Female-specific isoform
Methapyrilene toxicity	No toxicity	Toxicity	Toxicity dependent of CYP2C11 (Ratra *et al.*, 1998)
Phase II expression			
SULTs	3/14 genes expressed.	5/14 genes expressed.	11/14 genes expressed in liver.
UDPGTs	2/8 hepatic genes expressed.	5/8 hepatic genes expressed.	8/8 genes detectable in liver.
GSTs	8/21 genes expressed.	15/21 genes expressed.	18/21 genes detectable in liver.
CYP1A family expression and activity			
AhR expression	+	+	
CYP1A1 expression	+	+	
CYP1A2 expression	−	−	Genes disrupted in B-13 cells.
CYP1B1 expression	+	−	
AhR-dependent CYP1A1 induction (β-NF)	Induction	Induction	
EROD activity induction (β-NF)	Induction	Induction	
Benzo[α]pyrene genotoxicity	@ 100µM	@ 25µM and higher	
CYP2B family expression and activity			
CAR expression	−	+	
CYP2B1 expression	−	+	
CAR-dependent CYP2B1 induction (PB, TCPOBOP, DEX)	None	Induction	Restricted to mRNA induction.
PROD activity induction (PB)	None	None	Very low levels of increase with PB and DEX, likely associated with other P450s. CYP2B translation appears to be absent in B-13/H cells.
Cyclophosphamide genotoxicity	−	−	Observed at 200µM in B-13/H cells only—likely associated with activation by other CYPs
CYP3A family expression and activity			
PXR expression	−	+	
CYP3A1 expression	−	+	
CYP3A2 expression	−	+	
PXR-dependent CYP3A1 induction (PB, DEX, PCN)	None	Induction	
Luciferin-IPA activity induction (PB, DEX, PCN)	None	Induction	
Aflatoxin B1 genotoxicity	None*	@ at least 100nM after induction with DEX.	*Treatment of B-13 cells with inducing high levels of DEX likely resulted in partial differentiation of cells to B-13/H cells.
Transporter function			
Abcb11 (BSEP)	None	Detectable	
Abcb1b (p-glycoprotein)	Detectable	Detectable	
Abcc (MRP1-6)	Detectable	Detectable	
Abcg2 (BCRP)	Detectable	Detectable	

−, no/low expression; +, expressed.

Recent work from this laboratory has demonstrated using PCR-based approaches, cytogenetics, and fluorescent *in situ* hybridization techniques that the B-13 cell was derived from a male rat (Fairhall *et al.*, 2013a). The data in this paper show that when converted to B-13/H cells, the major male-specific CYP2C11 gene is expressed at levels at least 100 times higher than the major female-specific CYP2C12 gene. These data suggest that the B-13/H cell has maintained to a significant degree, the sexually dimorphic hepatic expression of CYPs.

The sexual dimorphism of several hepatic CYPs has been shown to be regulated by neonatal sex hormone imprinting the cyclical growth hormone secretion from the pituitary (Morgan *et al.*, 1985; Sharma *et al.*, 2012; Waxman and Holloway, 2009). Thus, *in vivo*, it is thought that the distinct male and female cyclical serum growth hormone patterns give rise to the pattern of expression of the male (eg, CYP2C11) and female forms (eg, CYP2C12). CYP2C11 is the major hepatic CYP in male rat liver and is reported to be specifically expressed in the liver (Thangavel *et al.*, 2007). In our hands, CYP2C11 mRNA was not detectable in pancreas, spleen, heart, brain, or lung. Only the kidney expressed detectable levels of CYP2C11 mRNA, at < 1% of hepatic levels as determined by quantitative RT-PCR (data not shown). Expression of the CYP2C11 protein at levels similar to male rat liver and the potent toxicity of methapyrilene in B-13/H cells serve to emphasize the quantitative nature of conversion that occurs as B-13 cells differentiate into B-13/H cell.

The data in this paper suggest that there may be an additional epigenetic mechanism of regulation of CYP at the level of the liver because growth hormone levels in culture (present within the serum component of the culture medium) were not modulated into a cyclical concentration pattern to mimic the male rat pattern observed *in vivo*. Remarkably, despite being generated at least 18 years ago (Mashima *et al.*, 1996), B-13 cells have retained this epigenetic control of CYP2C11 expression. B-13 cells may therefore be an important tool with which to investigate this aspect of sexually dimorphic expression of hepatic gene expression.

In terms of screening for drug and chemical toxicity and genotoxicity, B-13/H cells may have considerable utility given their ease of use. Both B-13 and B-13/H cells expressed CYP1A1 and B-13/H cells responded similarly to rat hepatocytes with respect to AhR-dependent induction of CYP1A1, resulting in functional CYP1A1 protein expression. Accordingly, dose-dependent increases in DNA damage were detectable in B-13/H cells after exposure to benzo[α]pyrene, after pretreatment with the AhR agonist β-NF.

B-13/H cells expressed low levels of CAR and induced the CAR-responsive gene CYP2B1 to high levels in response to treatment with CAR activators in a manner more qualitatively reflective of the rat liver *in vivo* than primary rat hepatocytes. However, functional CYP2B activity was low and increased minimally by CAR activator treatment, with only relatively high concentrations of cyclophosphamide capable of inducing DNA damage in B-13/H cells. Culturing B-13/H cells on collagen or collagen sandwiches, which have been reported to potentiate CYP2B induction response in primary rat hepatocytes (De Smet *et al.*, 2001; Kocarek *et al.*, 1994), did not result in a more effective functional CYP2B induction (data not included), and therefore the reason why CYP2B1 mRNA was not efficiently translated into functional protein is currently unclear. One potential explanation could be that the CYP2B induction response is more sensitive to limited heme availability (Acharya *et al.*, 2010), although we have demonstrated that B-13/H cells synthesize heme and are capable of generating CYP spectrophotometrically detectable in its reduced state when bound with carbon monoxide (which requires heme incorporation) (Fairhall *et al.*, 2013a). Thus, under the culture conditions employed, B-13 cells remain limited in their ability to identify CYP2B-dependent toxicity and genotoxicity. However, they may be a tractable model in which to study the mechanism of CYP2B induction at the transcriptional level. B-13 cells may also be an effective model system in which to identify regulators of CYP2B translation.

B-13/H cells expressed PXR and induced the PXR-responsive gene CYP3A1 gene to high levels in response to treatment with PXR activators. In contrast to CYP2B enzymes, CYP3A enzymes were significantly functional and activated aflatoxin B1 to a DNA-damaging species.

Gene expression associated with phase II metabolism was also examined in B-13 and B-13/H cells. RT-PCR screening suggests that the expression of a number of genes is increased as the B-13 cells transdifferentiate into B-13/H cells. However, it remains to be seen whether these changes result in the expression of functional phase II activity. Overall, however, the data suggest that B-13/H cells may be a valuable and readily applicable model for studying and/or screening for hepatoxicity. In terms of genotoxicity, B-13/H cells may be limited to comet assays (albeit limited currently to CYP1A- and CYP3A-activated compounds). As observed with primary rat hepatocytes, B-13/H cells appear to be refractive to mitogens in culture, although the reasons for this remain unclear because hepatocytes readily undergo mitosis *in vivo* (Wallace *et al.*, 2010a). However, if proliferation can be induced in B-13/H cells, then B-13/H cells may also be suitable for testing chemicals for clastogenicity.

Transporters play a key role in determining the toxicity of drugs and chemicals, although their study has been limited because their expression is rapidly lost in primary hepatocytes (Borlak and Klutcka, 2004; Tchaparian *et al.*, 2011). Functional studies are also limited by the fact that transporter expression is localized to different membrane compartments of the hepatocyte. *In vivo*, the hepatocyte is polarized and this is challenging to replicate *in vitro* and may impact on transporter expression and function.

B-13/H cells upregulated the expression of all transporter mRNAs (relative to B-13 cells) and expressed several at levels similar to the liver *in vivo*. However, many transporters were expressed at low levels in B-13/H cells compared with those seen *in vivo*. For example, BSEP and Na+-taurocholate cotransporting polypeptide—transporters involved in the efflux and influx of bile acids, respectively—were poorly expressed in both B-13 and B-13/H cells. Although several transporters were expressed at relatively low levels in B-13/H cells, efflux assays indicated that the transporters were detectably functional in these cells. The accessible 2 dimensional culture system employed in these studies may not be optimal for all hepatic functions. Sandwich culture has been shown to promote the formation of bile canaliculi (Swift *et al.*, 2010) and may therefore enhance transporter function in B-13/H cells. Future studies using 3D culture formats and sandwich culture with a variety of extracellular matrix formulations may be required to increase transporter gene expression in B-13/H cells.

Because B-13 cells are rat cells, species differences could limit extrapolation of toxicity data to man. However, introduction of human drug-metabolizing enzymes and drug transporters could be used to humanize the line. The humanization of B-13 cells with human CYP1A2 demonstrates proof of concept that the B-13 cell line can be readily modified in this way.

To our knowledge, the generation from a progenitor or stem cell of hepatocyte-like cells with functions in many cases at a quantitatively similar to levels in primary hepatocytes has not been demonstrated for any other cell line, with the exception of HepRG cells (Andersson, 2010). Given the simple culture conditions and the requirement for a single hormone treatment to convert B-13 cells to B-13/H cells, B-13 cells will have considerable utility in the study of hepatotoxicity and genotoxicity studies. Furthermore, the replacement of primary hepatocytes with B-13/H cells would significantly reduce the number of animals used for hepatocyte generation.

## SUPPLEMENTARY DATA

Supplementary data are available online at http://toxsci.oxfordjournals.org/.

## FUNDING

This work was supported by grants from the National Centre for the Replacement, Refinement and Reduction of Animals in Research (NC3Rs, grant NC/K500471/1; http://www.nc3rs.org.uk/), the EPSRC Knowledge Transfer Account (KTA), the Alternatives Research and Development Foundation (http://www.ardf-online.org/), and by European Commission FP7 program grant’D-Liver’ (EC Contract No. 287596; http://www.D-LIVER.eu/). P.M.E.P. is supported by a studentship award from the NC3Rs and a short term travelling fellowship from the In Vitro Toxicology Society (http://www.ivts.org.uk/site/).

## Supplementary Material

Supplementary Data
