# Supplementary material for: Utility of B-13 Progenitor-Derived Hepatocytes in Hepatotoxicity and Genotoxicity Studies
Source: Toxicol Sci. 2013 Nov 13;137(2):350–70. doi: 10.1093/toxsci/kft258 (PMC3908725; doi:10.1093/toxsci/kft258)
Supplement: Supplementary Data [file supp_137_2_350__index.html]

Utility of B-13 Progenitor-Derived Hepatocytes in Hepatotoxicity and Genotoxicity Studies — Utility of B-13 Progenitor-Derived Hepatocytes in Hepatotoxicity and Genotoxicity Studies — Utility of B-13 Progenitor-Derived Hepatocytes in Hepatotoxicity and Genotoxicity Studies — Supplementary Data 

# Utility of B-13 Progenitor-Derived Hepatocytes in Hepatotoxicity and Genotoxicity Studies

## Supplementary Data

Data files

**Files in this Data Supplement:**

- Supplementary Data - Supplementary Data
- Supplementary Data - Supplementary Data
- Supplementary Data - Supplementary Data
- Supplementary Data - Supplementary Data
- Supplementary Data - Supplementary Data
- Supplementary Data - Supplementary Data
