# Supplementary figures and images for: Utility of B-13 Progenitor-Derived Hepatocytes in Hepatotoxicity and Genotoxicity Studies
Source: Toxicol Sci. 2013 Nov 13;137(2):350–70. doi: 10.1093/toxsci/kft258 (PMC3908725; doi:10.1093/toxsci/kft258)

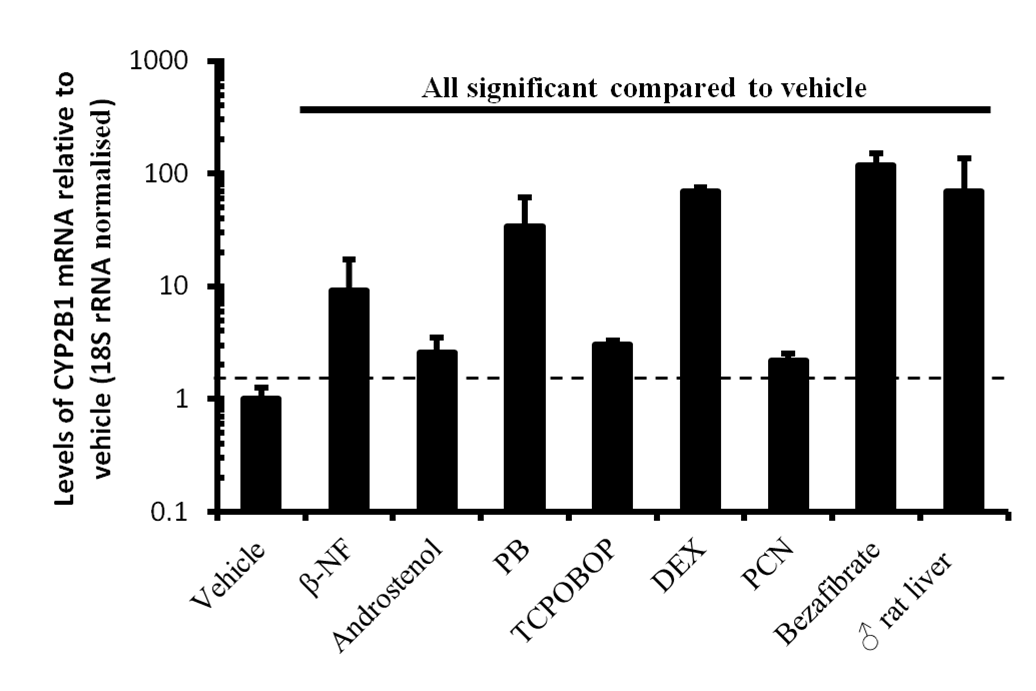

Supplement: Supplementary Data [file supp_kft258_toxsci_13_0672_File012.tif]

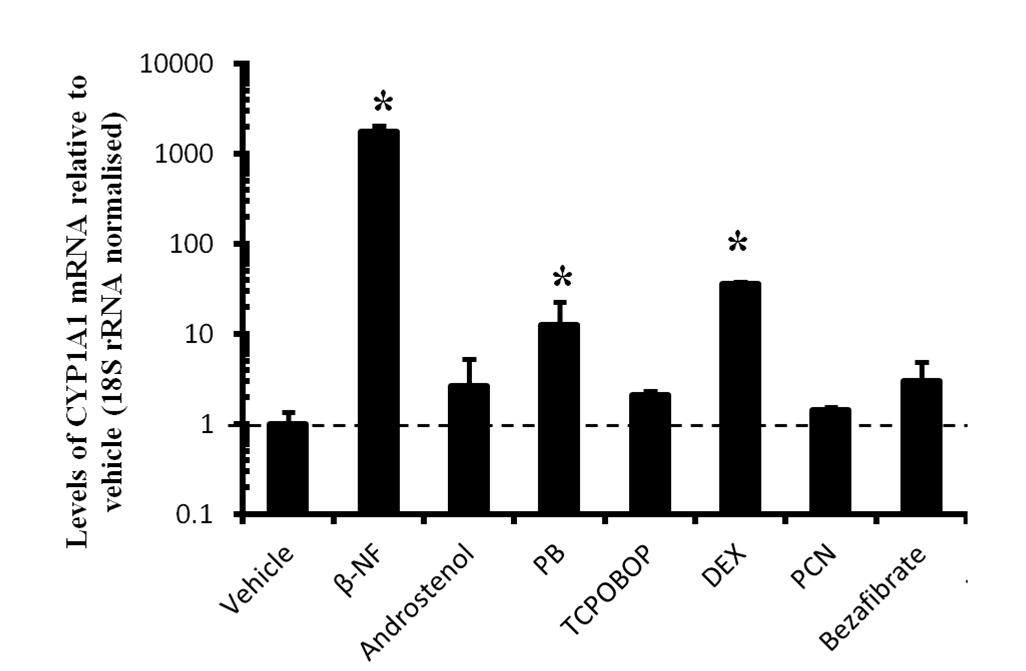

Supplement: Supplementary Data [file supp_kft258_toxsci_13_0672_File010.tif]

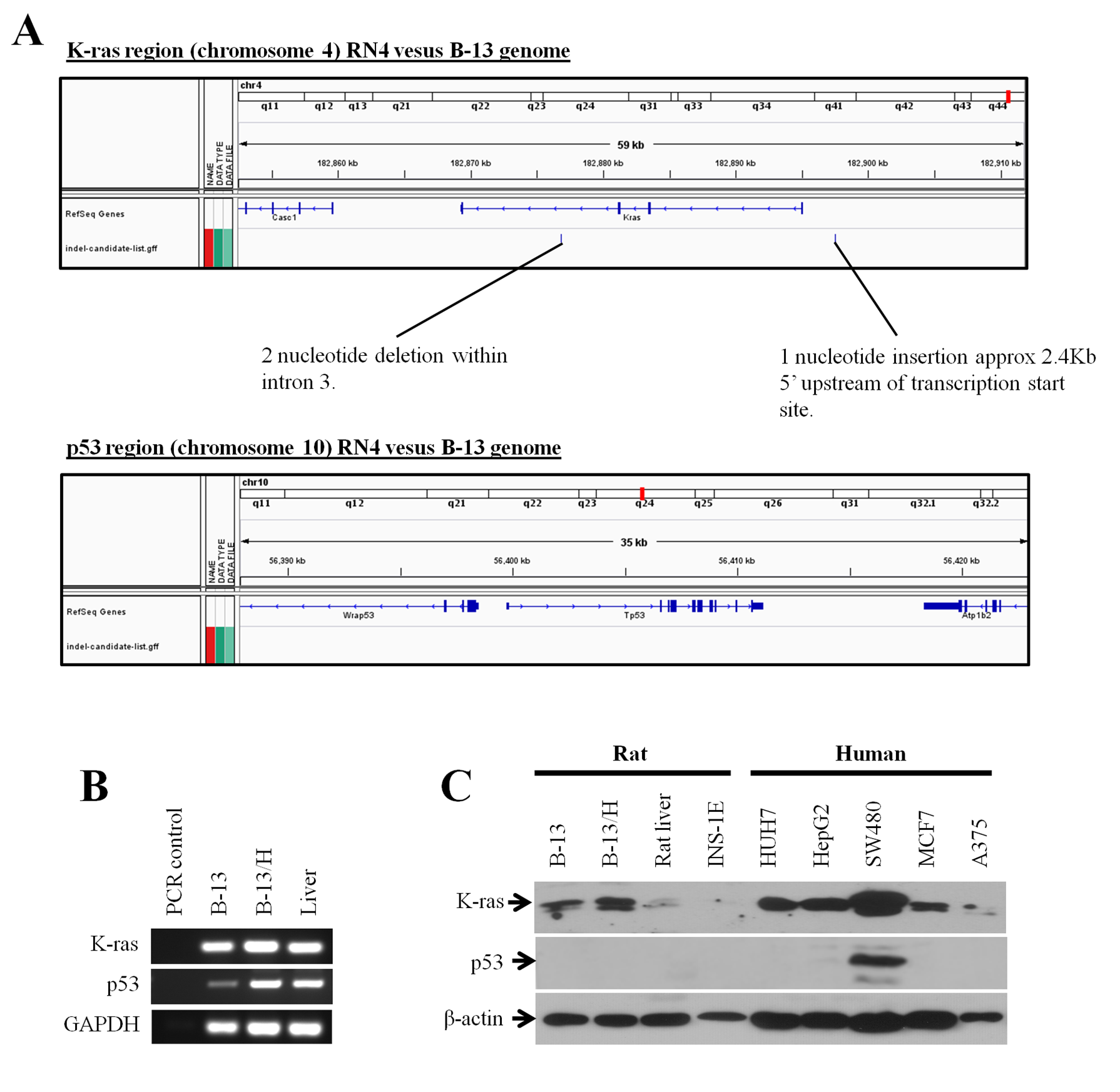

Supplement: Supplementary Data [file supp_kft258_toxsci_13_0672_File014.tif]

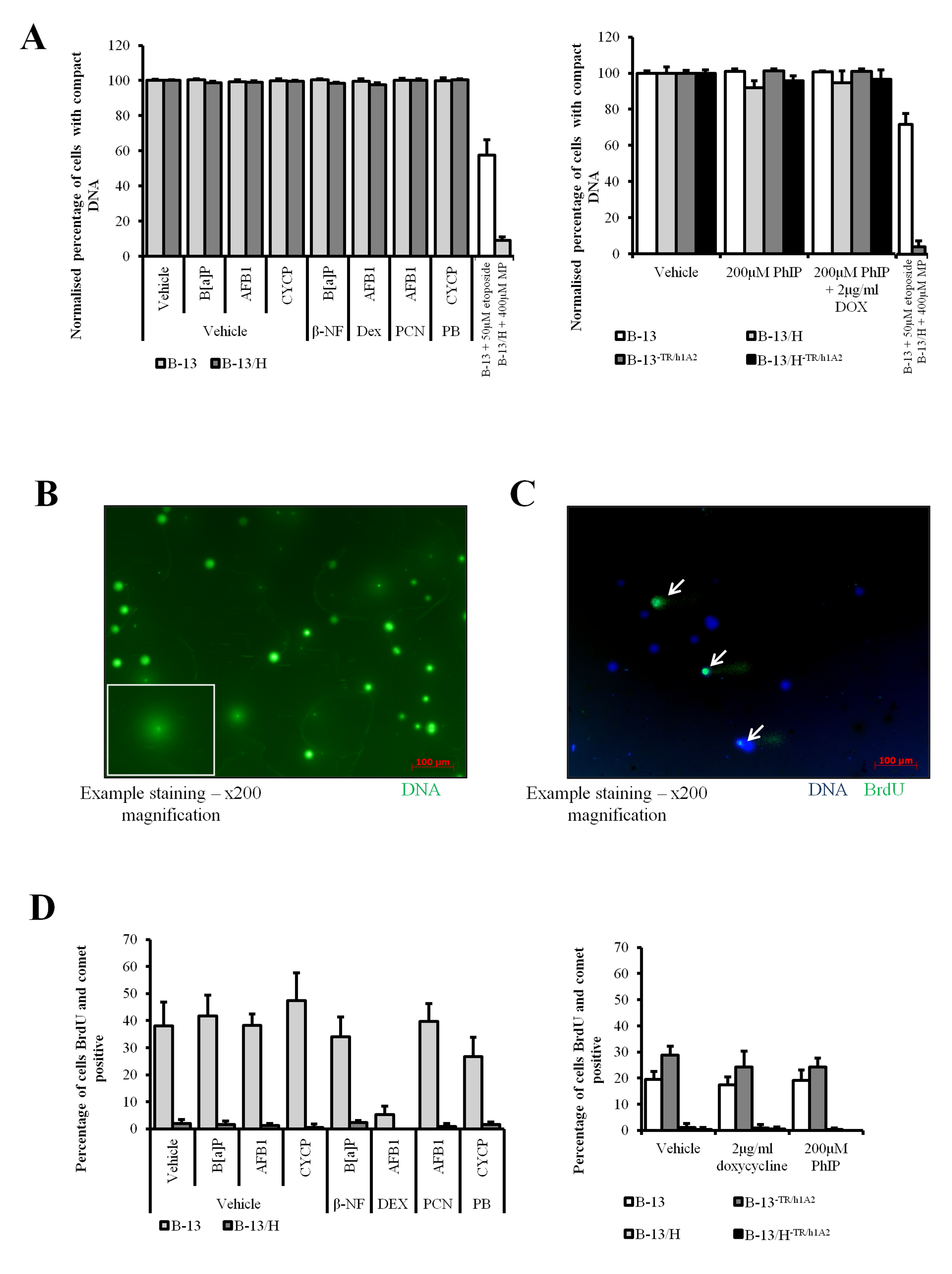

Supplement: Supplementary Data [file supp_kft258_toxsci_13_0672_File011.tif]

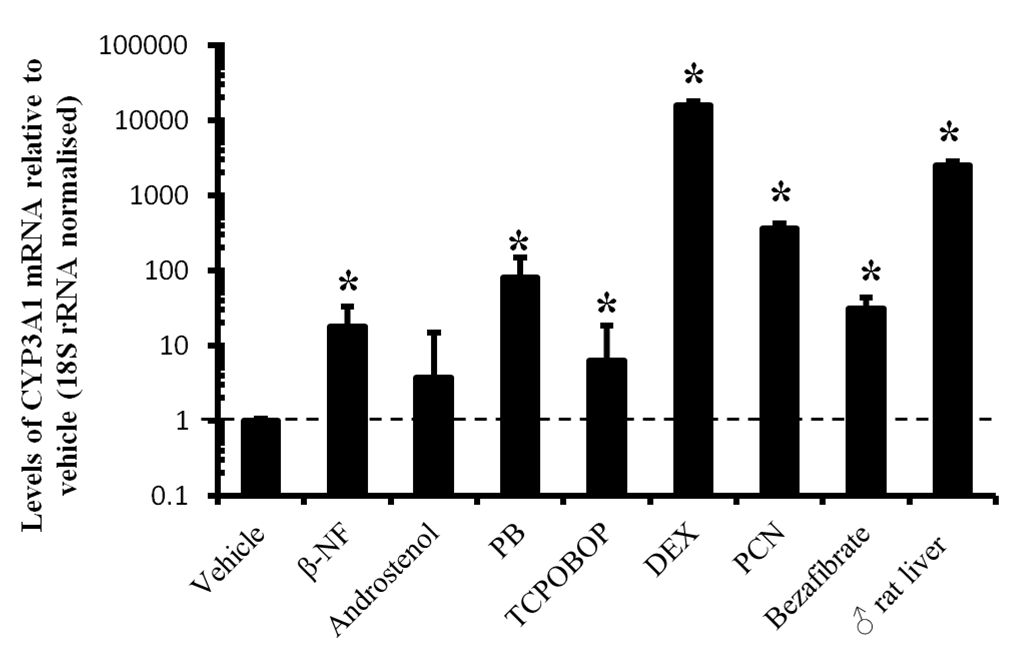

Supplement: Supplementary Data [file supp_kft258_toxsci_13_0672_File013.tif]
