# Supplementary material for: Utility of B-13 Progenitor-Derived Hepatocytes in Hepatotoxicity and Genotoxicity Studies
Source: Toxicol Sci. 2013 Nov 13;137(2):350–70. doi: 10.1093/toxsci/kft258 (PMC3908725; doi:10.1093/toxsci/kft258)
Supplement: Supplementary Data [file supp_kft258_Supplementary_Figures.docx]

**Supplementary Figure 1:** Quantitative RT-PCR for CYP1A1 mRNA in primary rat hepatocytes in response to indicated inducer treatments. Data are mean and standard deviation of 3 separate determinations from the same experiment, typical of 3 separate experiments. *Significantly different from vehicle only treated cells.

**Supplementary Figure 2:** Appearance of DNA damage in B-13/H cells is not associated with cytotoxicity in response to genotoxic agent treatment nor with DNA synthesis. **A)** Percentage of B-13 and B-13/H cells with intact nuclei after treatment for 24 hours with the highest genotoxin dose employed. Results are the mean and standard deviation from 4 fields of view, with at least 100 individual cells counted in total, from a single experiment, typical of three separate experiments. **B)** Photomicrograph of vehicle treated B-13 cells at x100 magnification and inset – zoomed in image of etoposide-treated apoptotic cell showing diffuse DNA. **C)** B-13 and B-3/H cells were incubated for 24 hours with the highest genotoxin dose employed. Two hours prior to harvesting, 15µM BrdU was added to cultures – typical results at x200 magnification showing B-13 cells (which are proliferative) positive for both BrdU incorporation and an apparent comet. **D**) Results from B-13 and B-13/H cells treated as in C**.** Data are the mean and standard deviation of BrdU-positive cells also showing comets after treatment with the indicated compounds calculated from 5 fields of view, with at least 300 individual cells counted in total. Results are from a single experiment, typical of 3.

**Supplementary Figure 3:** Quantitative RT-PCR for CYP2B1 mRNA in primary rat hepatocytes in response to indicated inducer treatments. Data are mean and standard deviation of 3 separate determinations from the same experiment, typical of 3 separate experiments.

**Supplementary Figure 4:** Quantitative RT-PCR for CYP3A1 mRNA in primary rat hepatocytes in response to indicated inducer treatments. Data are mean and standard deviation of 3 separate determinations from the same experiment, typical of 3 separate experiments. *Significantly different from vehicle only treated cells.

**Supplementary Figure 5**: K-ras and p53 gene expression in B-13 and B-13/H cells. A) Schematic diagram of B-13 K-ras and p53 genomic regions indicating insertions and deletions used in PCR amplification. B) RT-PCR for K-ras and P53 mRNA. C) Western blot for K-ras and p53 in B-13, B-13/H, rat liver and a variety of rat (INS-1E) and human cancer cell lines as indicated (10 µg cell protein/lane). Data are typical of 3 separate determinations with B-13 and B-13/H cells.
